# Stimulation of Na^+^/H^+^ Exchanger Isoform 1 Promotes Microglial Migration

**DOI:** 10.1371/journal.pone.0074201

**Published:** 2013-08-21

**Authors:** Yejie Shi, Hui Yuan, Dong Kim, Vishal Chanana, Akemichi Baba, Toshio Matsuda, Pelin Cengiz, Peter Ferrazzano, Dandan Sun

**Affiliations:** 1 Department of Neurology, University of Pittsburgh, Pittsburgh, Pennsylvania, United States of America; 2 Neuroscience Training Program, University of Wisconsin, Madison, Wisconsin, United States of America; 3 Department of Neurological Surgery, University of Wisconsin, Madison, Wisconsin, United States of America; 4 Waisman Center, University of Wisconsin, Madison, Wisconsin, United States of America; 5 Graduate School of Pharmaceutical Sciences, Osaka University, Osaka, Japan; Xuzhou Medical college, China

## Abstract

Regulation of microglial migration is not well understood. In this study, we proposed that Na^+^/H^+^ exchanger isoform 1 (NHE-1) is important in microglial migration. NHE-1 protein was co-localized with cytoskeletal protein ezrin in lamellipodia of microglia and maintained its more alkaline intracellular pH (pH_i_). Chemoattractant bradykinin (BK) stimulated microglial migration by increasing lamellipodial area and protrusion rate, but reducing lamellipodial persistence time. Interestingly, blocking NHE-1 activity with its potent inhibitor HOE 642 not only acidified microglia, abolished the BK-triggered dynamic changes of lamellipodia, but also reduced microglial motility and microchemotaxis in response to BK. In addition, NHE-1 activation resulted in intracellular Na^+^ loading as well as intracellular Ca^2+^ elevation mediated by stimulating reverse mode operation of Na^+^/Ca^2+^ exchange (NCX_rev_). Taken together, our study shows that NHE-1 protein is abundantly expressed in microglial lamellipodia and maintains alkaline pH_i_ in response to BK stimulation. In addition, NHE-1 and NCX_rev_ play a concerted role in BK-induced microglial migration via Na^+^ and Ca^2+^ signaling.

## Introduction

Microglia are resident macrophages and ubiquitously distributed in the central nervous system (CNS). They serve as sensors for CNS homeostasis disruption and can be activated under various pathological conditions [1]. Microglial activation includes morphological changes, proliferation, and migration. One of the earliest microglial responses to brain injury is its migration to the site of injury or inflammation [2]. Extracellular ATP and ADP released from ischemic and traumatic brain tissues can stimulate microglial migration [3]. Injury to the CNS tissues also releases other chemotactic factors including morphine [4], epidermal growth factor [5], cannabinoids [6] and bradykinin [7], which stimulate microglial migration to the site of injury. Microglia have been demonstrated to move along the chemokine gradients in *in vitro* and *in vivo* injury models for experimental autoimmune encephalitis, Alzheimer’s disease, or cerebral ischemia [8].

Directed cell movement is a multi-step process requiring an initial spatial polarization that is established by asymmetric stimulation of Rho GTPase, phosphoinositides (PIs), and actin polymerization [9]. Dynamic surface protrusions, such as lamellipodia and filopodia, are fundamental determinants of cell motility and migration [10]. Accumulation of filamentous actin (F-actin) is considered as the driving force for membrane protrusion [11]. Formation of lamellipodia and ruffles are likely the rate-limiting events occurring during cell motility [12]. However, how lamellipodia and filopodia are formed and regulated during microglial migration remains poorly understood.

In motile mammalian fibroblasts, increased activity of the ubiquitous actin-binding proteins cofilin requires an alkaline intracellular pH (pH_i_) environment for the assembly of new actin filaments [13]. Therefore, Na^+^/H^+^ exchanger isoform 1 (NHE-1) has been suggested to play a role in regulation of cofilin activity and acceleration of F-actin treadmilling in lamellipodia or filopodia of fibroblasts [13]. NHE-1 also serves as a focal contact and anchors the actin cytoskeleton to the plasma membrane through its c-terminal binding to the ezrin/radixin/moesin (ERM) protein family [14]. Therefore, NHE-1 may modulate cell migration via interacting with cytoskeleton proteins. However, whether NHE-1 interacts with cytoskeletal proteins and plays a role in microglial migration remains unknown.

In the current study, we investigated whether NHE-1 is involved in regulation of microglial migration in cultured primary or murine BV2 microglial cells in response to bradykinin (BK) stimulation. Our study clearly demonstrated that NHE-1 protein is co-localized with cytoskeletal protein ezrin in microglial lamellipodia and stimulates microglial migration by regulating alkaline pH_i_ and Na^+^ and Ca^2+^ signaling.

## Materials and Methods

### Ethics Statement

All animal experiments were approved by the University of Pittsburgh Institutional Animal Care and Use Committee and performed in accordance with the National Institutes of Health Guide for the Care and Use of Laboratory Animals.

### Materials

Hanks balanced salt solution (HBSS) and penicillin-streptomycin solution were from Mediatech Cellgro (Manassas, VA, USA). Alexa Fluor® 488 phalloidin, 1,2-bis(o-aminophenoxy) ethane-N,N,N',N'-tetraacetic acid (BAPTA) AM, 2’,7’-bis-(2-carboxyethyl)-5-(and-6)-carboxyfluoreescein (BCECF) AM, calcein AM, 4',6-diamidino-2-phenylindole (DAPI), Dulbecco’s Modified Eagle Medium (DMEM), DMEM-F12, fetal bovine serum (FBS), Fluo-4 AM, Fura-Red AM, goat anti-mouse Alexa Fluor® 488-conjugated IgG, goat anti-rabbit Alexa Fluor® 546-conjugated IgG, Lipofectamine® RNAiMAX Transfection Reagent, Opti-MEM®, sodium-binding benzofuran isophthalate (SBFI) AM, To-pro-3 iodide and Versene were from Invitrogen (Carlsbad, CA, USA). Bradykinin, Cytochalasin D, gramicidin, monensin and nigericin were purchased from Sigma-Aldrich (St. Louis, MO, USA). Mouse anti-NHE-1 monoclonal antibody was purchased from Santa Cruz Biotechnology (Santa Cruz, CA, USA). Rabbit anti-ezrin polyclonal antibody, rabbit anti-ezrin/radixin/moesin polyclonal antibody, rabbit anti-phospho-ezrin (Thr567) / radixin (Thr564) / moesin (Thr558) polyclonal antibody, and rabbit anti-glyceraldehyde-3-phosphate dehydrogenase (GAPDH) monoclonal antibody were purchased from Cell Signaling (Danvers, MA, USA). Rabbit anti-Na^+^/Ca^2+^ exchanger isoform 1 (NCX-1) polyclonal antibody was purchased from Swant (Marly, Switzerland). HOE 642 was a kind gift from Aventis Pharma (Frankfurt, Germany). SEA0400 was a kind gift from Taisho Pharmaceutical Co. Ltd. (Omiya, Saitama, Japan).

### Primary microglial culture

Mixed primary glial cultures were established from mouse brains (Black Swiss; Taconic Farms, Inc., Hudson, NY, USA) as described previously [15] with minor modifications. Briefly, dissociated cells from brain tissues of 1-3 day-old mice (male and female) were collected. The cell suspension was centrifuged and resuspended in the DMEM-F12 complete medium (containing 10% FBS, 100 U/mL penicillin, and 100 µg/mL streptomycin). The cells were seeded in poly-D-lysine-coated culture flasks and maintained at 37°C with 5% CO_2_ + 95% air in an incubator (Thermo Scientific; Model: 3110) and refed three times a week. Following 10-14 days of incubation, microglia were removed from the astroglial layer by shaking the flasks on an orbital shaker (Labnet; Model: Orbit P4) for 2 h at 250 g. The microglial pellets were resuspended in the complete DMEM medium and seeded on the poly-D-lysine coated glass coverslips in 6-well plates (4-5 × 10^5^ cells/well) or on 35 mm glass bottom dishes (4-5 × 10^5^ cells/dish). Experiments were performed on day 1-3 after seeding.

Na^+^/Ca^2+^ exchanger isoform 1 heterozygous (NCX-1^+/-^) mouse colony in SV129/Black Swiss background was maintained as described previously [16]. For NCX-1^+/-^ cultures, tail biopsy was obtained from each pup and polymerase chain reaction (PCR) analysis of DNA was performed to determine the genotype of each culture as described previously [16].

### BV2 microglial culture

Murine BV2 microglial cells were cultured in T25 flasks in the complete DMEM-F12 medium. Cell cultures were maintained at 37^°^C with 5% CO_2_ + 95% air and subcultured (1:4) every 2 days by gently removing the cells with Versene. Cells were seeded on poly-D-lysine coated glass coverslips in 6-well plates (10^5^ cells/well) or on 35 mm glass bottom dishes (10^5^ cells/dish). Experiments were performed on day 1 after seeding. Cultures of passage 18 or lower were used in this study.

### Cell motility measurement

Microglial cells seeded on 35 mm glass bottom dishes were placed in a stage top incubator at 37°C with 5% CO_2_ + 95% air (model: TIZ, Tokai Hit; Shizuoka-ken, Japan). Motility of microglia was monitored under the 40 × oil immersion objective lens with a time-lapse video microscope system (the Nikon TiE 300 inverted epifluorescence microscope with perfect focus) and MetaMorph software (Molecular Devices, Sunnyvale, CA, USA). Time-lapse DIC images were acquired in 1-min intervals for 60 min either under serum-free DMEM control condition or in the presence of BK, HOE 642, SEA 0400, or BAPTA AM. Images were analyzed by ImageJ software (National Institute of Health, USA) and cell tracking was performed using the Manual Tracking plugin. Total distance traveled was determined by tracking the movement of the cell gravity center, and its coordinates were used to calculate the distances.

In some studies, BV2 or primary microglia were incubated with 0.5 µM calcein AM for 30 min at 37°C. Calcein fluorescence in a cell was monitored under a 40 × objective lens using a FITC filter set (excitation 480 nm, emission 535 nm, Chroma Technology, Rockingham, VT, USA).

### Image analysis for microglial lamellipodial morphological changes

Analysis for morphological changes of lamellipodia in microglia was performed using the ImageJ software. To measure fluctuations in lamellipodial area through time, the lamellipodia was traced manually every 2 min and area calculated with the region of interest (ROI) manager. Lamellipodial length was defined as the furthest point of lamellipodia to cell body and quantified using the Straight Line tool. Lamellipodial persistence was defined as the time of lamellipodial extension along the moving direction before it retracts, and was calculated using the kymograph plugin as described before [12]. Protrusion rate was calculated as lamellipodial length divided by persistence (µm/min). Lamellipodial change was calculated by dividing the mean lamellipodia area over time by the corresponding persistence (µm^2^ /min). A minimum of five cells were analyzed from each experiment and an average calculated.

### pH_i_ measurement

pH_i_ measurement in microglia was performed as described previously [17]. Briefly, microglial cultures grown on coverslips were incubated with 0.5-2.5 µM BCECF AM at 37 °C for 30 min. The coverslips were placed in a temperature-controlled (37 °C) open-bath imaging chamber containing HCO_3_^-^-free HEPES-MEM [17]. On the stage of the Nikon TiE 300 inverted epifluorescence microscope, the cells were visualized with a 40 × oil immersion objective lens and excited every 20 s at 440 and 490 nm, and the emission fluorescence at 535 nm was recorded. Images were collected using a Princeton Instruments MicroMax CCD camera and analyzed with MetaFluor image-processing software (Molecular Devices, Sunnyvale, CA, USA) as described previously [17].

### Intracellular Ca^2+^ measurement

Intracellular Ca^2+^ concentration ([Ca^2+^]_i_) elevation was detected by ratiometric imaging of Fluo-4 and Fura-Red fluorescent signals as described before [18]. Cells grown on coverslips were incubated with 7.5 µM Fluo-4 AM and Fura-Red AM (with 0.01% pluronic acid and 0.2% DMSO) in the HEPES-MEM buffer at room temperature for 30 min. Cells were washed and then incubated at 37°C for another 30 min to allow deesterification prior to the measurement. Under the similar setting described in the pH_i_ measurement, cells were excited at 490 nm and the emission fluorescence at both 530 nm (Fluo-4) and 660 nm (Fure-Red) wavelengths collected. Images were collected every 5 min for 40-70 min and analyzed with the MetaFlour image-processing software. The changes of [Ca^2+^]_i_ in single cells were determined by calculating fluorescence intensity ratios of Fluo-4/Fura-Red.

### Intracellular Na^+^ measurement

Intracellular Na^+^ concentration ([Na^+^]_i_) was measured in primary microglia with the fluorescent dye SBFI AM as described previously [19]. Cultured microglia grown on coverslips were loaded with 10 µM SBFI AM plus 0.02% pluronic acid at 37°C for 60 min. Using the Nikon TiE 300 inverted epifluorescence microscope and a 40 × oil immersion lens, cells were excited at 345 and 385 nm, and the emission fluorescence at 510 nm was recorded. Images were collected every 5 min for 35 min to determine Na^+^_i_ levels, and the 345/385 ratios were analyzed with the MetaFluor image-processing software. Absolute [Na^+^]_i_ was determined for each cell as described before [19].

### Microchemotaxis assay

Chemotaxis of microglia was tested using a 24-well microchemotaxis Boyden chamber (8 µm pore size; BD Falcon, Sparks, MD, USA) as described before [5]. Microglial cells (10^5^/mL) in 100 µL of serum-free DMEM with or without 1 µM HOE 642 were added to the upper wells, and the lower wells contained either 700 µL serum-free DMEM or DMEM plus 300 nM BK. The chamber was incubated at 37°C and 5% CO_2_ in the incubator for 5 h. Cells remaining on the upper surface of the membrane were removed with cotton-tipped swabs. The membrane was rinsed with PBS and the migrated cells on the bottom surface of the membrane were fixed with 4% paraformaldehyde (PFA) and subjected to DAPI staining (2 µg/mL in PBS). Samples were excited at 358 nm with a xenon lamp and the emission fluorescence at 461 nm recorded with a Princeton Instruments MicroMax CCD camera attached to the Nikon TiE300 microscope using the MetaMorph software. Images of 5 random fields (220 µm × 165 µm) under the 40 × objective lens were captured. Migrated cells in all 5 fields were averaged to give a mean cell count for each experiment. Migration rate was expressed as percentage of control.

### Actin staining and quantification

Cells were fixed in 3.7% methanol-free formaldehyde solution in PBS for 10 min at room temperature. After rinsing in PBS, cells were extracted with 0.1% Triton X-100 in PBS for 5 min. After washing, cells were incubated with 1 unit of Alexa Fluor® 488 phalloidin in PBS/1% BSA (200 µL/coverslip) for 20 min. Cells were rinsed and incubated with To-pro-3 iodide (1:1000 in staining solution) for 15 min. Fluorescence images were captured with a Leica DMIRE2 inverted confocal laser-scanning microscope under the 40 × oil immersion objective lens (Leica Software, Mannheim, Germany). Samples were excited at 488 nm (argon/krypton) and 633 nm. The emission fluorescence was recorded at 512-548 nm and 650-750 nm, respectively. Actin quantification was performed using the ImageJ software on phalloidin-staining images. The lamellipodial area of each cell was traced with the ROI manager, and phalloidin fluorescent intensity was measured in each ROI. A minimum of five cells were analyzed from each experiment and an average intensity was calculated.

### Immunofluorescence staining and image analysis

Microglial cells grown on coverslips were fixed in 4% PFA in PBS. For Cytochalasin D (Cyt.D) treatment, 100 nM Cyt.D was added into the medium 20 h prior to fixation. After rinsing in PBS for 15 min, cells were incubated with a blocking solution (PBS/0.3% Triton X-100/5% goat serum) for 60 min. Cells were incubated with monoclonal mouse anti-NHE-1 antibody (1:100) and polyclonal rabbit anti-NCX-1 antibody (1:200) or polyclonal rabbit anti-ezrin antibody (1:200) diluted in the antibody diluting solution (PBS/0.3% Triton X-100/1% BSA) at 4°C overnight. After rinsing in PBS, cells were incubated with goat anti-mouse secondary antibody IgG conjugated to Alexa Fluor® 488 and goat anti-rabbit secondary antibody IgG conjugated to Alexa Fluor® 546 (1:200 dilution) for 2 h. Cells were then rinsed and incubate with To-pro-3 iodide (1:1000 in antibody diluting solution) for 15 min. Fluorescence images were captured with the Leica DMIRE2 inverted confocal laser-scanning microscope as described before [15]. Co-localization of NHE-1 and ezrin was analyzed using ImageJ software with the Colocalization Indices plug-in [20]. The Pearson’s correlation coefficient (CC) and overlap coefficient (OC) were used as indices of the frequency of co-localization between NHE-1 and ezrin as described previously [20]. For quantitative analysis, single optical sections were taken randomly from five to seven different regions on each coverslip. Cell roundness was calculated as 4π × cell area/(cell perimeter)^2^, with smaller value indicating more elongated morphology [21].

### RNA interference (RNAi) knockdown of NHE-1

Knockdown of NHE-1 protein expression in BV2 microglial cells was induced by the double-strand small interfering RNAs (siRNAs). SiRNA targeting NHE-1 (Sequence #1) was prepared as 3’-overhanged form (forward, 5’-CCACAAUUUGACCAACUUAtt-3’; reverse, 5’-UAAGUUGGUCAAAUUGUGGtc-3’; Invitrogen, Carlsbad, CA, USA). Stealth RNAi Negative Control (low GC content; Cat. No 12935-200; Invitrogen) was used as a control. Lipofectamine® RNAiMAX/siRNA complexes were formed in serum-free Opti-MEM® at 25°C for 5 min and added to 6-well plate. 250 µL of complexes in Opti-MEM containing 50 pmol siRNA and 7.5 µL Lipofectamine® RNAiMAX was added into each well. 10^5^ of BV2 cells in DMEM-F12 supplemented with 10% FBS was then added into each well to make a final volume of 2.5 mL per well and incubated at 37°C. Cultures were used at 48 h after transfection.

As shown in Figure **S1**, Sequence #1 showed excellent efficiency in selective knockdown of NHE-1 protein and did not affect expression of 2 other proteins NCX-1 and tERM, which are closely related in regulation of microglial migration. In contrast, Sequence #2 (forward, 5’-CGAAGAGAUCCACACACAGtt-3’; reverse, 5’-CUGUGUGUGGAUCUCUUCG tt-3’. Ambion) had minimum effects on reducing NHE-1 expression but downregulatied NCX-1 protein significantly (Figure **S1**). In light of the high efficiency and selectivity of NHE-1 siRNA sequence #1, we have used it in all experiments of this study.

### Immunoprecipitation and immunoblotting

BV2 cells were lysed in ice-cold lysis buffer (0.025 M Tris, 0.15 M NaCl, 0.001 M EDTA, 1% NP-40, 5% glycerol, pH 7.4) containing protease inhibitors and phosphatase inhibitor cocktail, and centrifuged at 11000 g for 10 min at 4°C. The supernatant was collected and protein content was determined by the bicinchoninic acid method. Protein samples (40-100 µg protein lysate) and pre-stained molecular mass markers (Bio-Rad) were denatured and separated on 10% sodium dodecyl sulfate polyacrylamide gel electrophoresis (SDS-PAGE). In addition, immunoprecipitation was conducted to examine interactions of NHE-1 and ezrin using the Pierce® Classic IP Kit (ThermoScientific, Rockford, IL, USA). Protein lysate samples (0.5 mg protein) were incubated with 20 µL of rabbit polyclonal antibody against ezrin at 4°C overnight. Immunocomplexes were mixed with 20 µL protein A/G beads (50% slurry) in a Pierce spin column and incubated for 2 h. The immunocomplexes were washed and dissociated from beads with the Laemmli sample buffer and heated at 95°C for 10 min. The resolved proteins and prestained molecular mass markers were separated on 10% SDS-PAGE, as described before [17]. The blots were incubated with rabbit anti-ezrin/radixin/moesin polyclonal antibody (1:1000), rabbit anti-phospho-ezrin (Thr567)/radixin (Thr564)/moesin (Thr558) polyclonal antibody (1:200), rabbit anti-GAPDH monoclonal antibody (1:5000), or rabbit anti-NHE-1 polyclonal antibody (1:500) overnight at 4°C. After rinsing, the blots were incubated with goat anti-rabbit or goat anti-mouse horseradish peroxidase-conjugated secondary IgG (1:2000) for 1 h. Bound antibody was visualized using the enhanced chemiluminescence assay (ThermoScientific, Rockford, IL, USA). Densitometric measurement of each protein band was performed using the Gel Analysis Tool in ImageJ.

### Statistical Analysis

Data are expressed as the mean ± SEM. Comparisons between groups were made by Student’s t-test or one-way ANOVA using the Bonferroni post-hoc test in the case of multiple comparisons (SigmaStat, Systat Software, Point Richmond, CA, USA), unless otherwise indicated. p < 0.05 was considered statistically significant. n values represent the number of independent cultures.

## Results

### BK-induced lamellipodial morphological changes of microglia depend on NHE-1 activity

**Figure 1:**
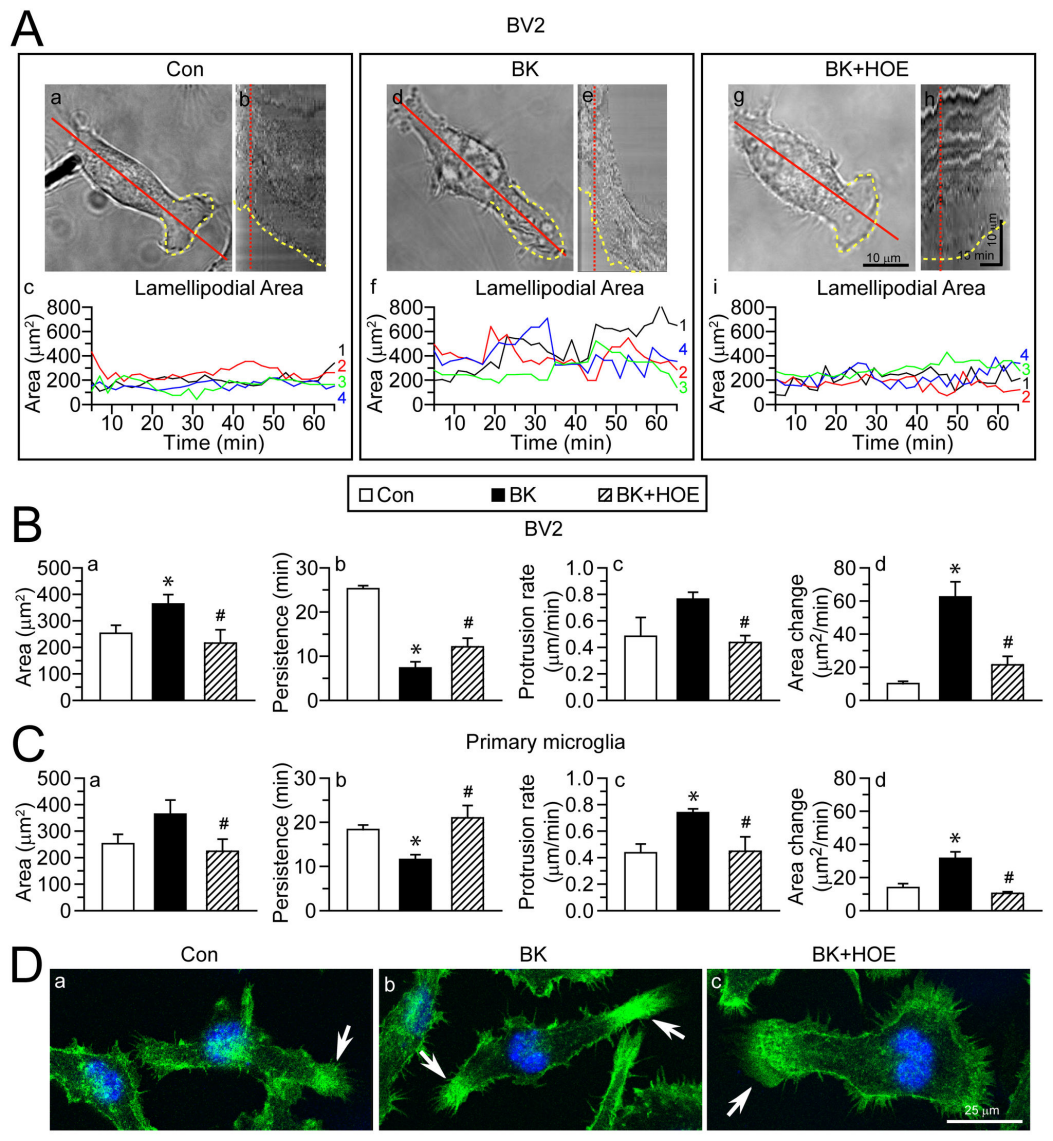
Inhibition of NHE-1 activity abolishes BK-induced lamellipodial dynamics in microglia. **A**. Lamellipodia formation in BV2 microglia were shown in the presence of control serum-free medium (**a**), 300 nM BK (**d**), or 300 nM BK plus 1 µM HOE 642 (**g**). *Yellow dashed line*: leading edge lamellipodial area. *Red solid line*: axis through which kymographs were generated. *Scale bar: 10 µm*. **b**, **e**, **h**: Kymographs under three conditions showing the morphological changes of the leading edge integrated by the 60-min time. *Red dotted line*: time point in kymograph corresponding to accompanying still graphs. *Horizontal scale bar*: 15 min. *Vertical Scale bar*: 10 µm. **c**, **f**, **i**: fluctuations in lamellipodial area of four representative cells (#1-4, in four different colors). **B**–**C**. Lamellipodial area (**a**), persistence (**b**), protrusion rate (**c**) and area change (**d**) were calculated in BV2 (**B**) or in primary microglia (**C**). Data are mean ± SEM. n = 4. * p < 0.05 vs. Con. # p < 0.05 vs. BK. **D**. Phalloidin staining images of actin fibers in BV2 cells. *Green*: Alexa Fluor® 488 phalloidin. *Blue*: To-pro-3 nuclear staining. *Arrow*: actin fibers at lamellipodia. *Scale bar*: 25 µm.

BK, a stimulant for microglial motility [7], was used to stimulate microglia in BV2 or primary microglia cultures. Figure **1A** shows that BV2 microglia exhibited formation of lamellipodial structure at leading edge (**a**, *yellow dashed line*). The lamellipodia was highly motile at the leading edge, which was reflected by the change in lamellipodial area in the kymograph (Figure **1A, b and c**). Upon BK stimulation, BV2 microglia displayed larger and more motile lamellipodia (Figure **1A, d and e**), with the area fluctuating from 141.7 µm^2^ to 823.2 µm^2^, compared to 41.7 µm^2^ to 431.1 µm^2^ under control condition (Figure **1A, f**). This was further confirmed by decreased lamellipodial persistence as calculated on the kymograph (Figure **1B, b**). As a result, the protrusion rate and area change of lamellipodia were significantly increased with BK stimulation (Figure **1B, c and d**). Interestingly, when NHE-1 activity was blocked with its potent inhibitor HOE 642, these changes were abolished. NHE-1 inhibition significantly reduced lamellipodial area and increased lamellipodial persistence, compared to the BK-stimulated BV2 cells (Figure **1A and B**). Similar results were obtained in primary microglia (Figure **1C**). In addition, lamellipodia formation was revealed by staining of F-actin at the end of experiment. Figure **1D** showed increased actin fluorescence intensity in lamellipodia at 60 min of BK stimulation (*arrow*). It was increased by 159.3 ± 8.2% of control (n = 4, p < 0.05) and reflected an increased actin polymerization in the lamellipodial area. In contrast, when NHE-1 activity was blocked with HOE 642, actin intensity in the lamellipodial area returned to the control level (95.4 ± 0.5% of control, n = 4). Taken together, these results indicate that in the presence of BK stimulation, microglia exhibited a more motile lamellipodia and increased cell motility. Interestingly, blocking NHE-1 activity abolished these changes.

In addition, inhibition of NHE-1 with HOE 642 treatment significantly reduced microglial motility under basal conditions and reduced total moving distance (Figure **S2**). These results suggest that NHE-1 plays an important role in microglial basal motility. This is consistent with the previous reports in fibroblasts [14].

### Interactions between NHE-1 and ERM in microglial lamellipodia

**Figure 2:**
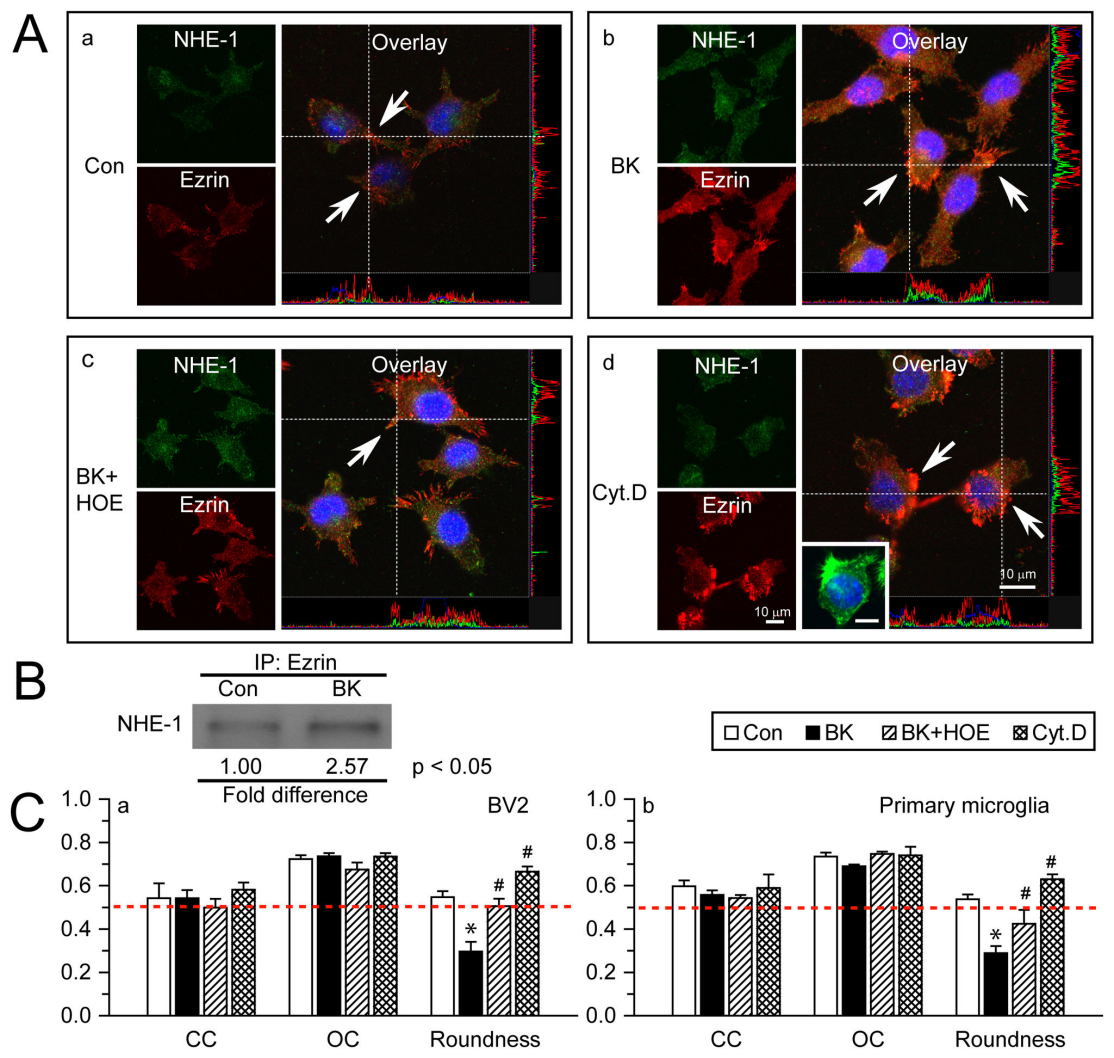
NHE-1 protein interacts with ERM in microglial lamellipodia. **A**. Representative immunostaining images of NHE-1 and Ezrin in control (**a**), BK-stimulated (**b**), BK and HOE 642-treated (**c**), or Cyt.D-treated BV2 (**d**). *Green*: NHE-1. *Red*: Ezrin. *Blue*: To-pro-3 nuclear staining. Co-localization of green and red signal and their intensity spectrum were shown along the white dashed lines on the overlay images. BV2 or primary microglia were stimulated with 300 nM BK for 1 h or 100 nM Cyt.D for 20 h. *Arrow*: co-localization of NHE-1 and ezrin at lamellipodia. **Inset in d**: Alexa Fluor® 488 phalloidin signal in cells treated with Cyt.D. *Scale bar in all panels*: 10 µm. **B**. Representative blot of immunoprecipitation of NHE-1 with anti-ezrin antibody in control or BK-stimulated BV2 was shown. n = 4. p < 0.05 Con. vs. BK. **C**. Summarized data of Pearson’s correlation coefficient (CC), overlap coefficient (OC) and cell roundness values for NHE-1 and ezrin signals in BV2 (**a**) and primary microglial cells (**b**). Data are mean ± SEM. n = 3. * p < 0.05 vs. Con. # p < 0.05 vs. BK. *Red dashed line*: reference line of value 0.5.

Given the importance of NHE-1 in lamellipodia formation in response to BK stimulation, we further investigated NHE-1 expression and its interactions with other cytoskeletal proteins. ERM proteins ezrin, radixin, and moesin are a group of actin-binding proteins that facilitates the link between the actin cytoskeleton and the plasma membrane [22,23]. NHE-1 has been shown to serve as focal contacts and anchors of the actin cytoskeleton to the plasma membrane through its direct binding of ERM proteins at the cytoplasmic C-terminal in fibroblasts [14]. Therefore, we examined expression of NHE-1 protein and ezrin in microglial cells. Under control conditions, NHE-1 and ezrin were largely co-localized in lamellipodia, as reflected by the overlap of green and red immunostaining signals (Figure **2A** **, a**, *arrow*). The Pearson’s correlation coefficient (CC) was 0.54 ± 0.07 and the overlap coefficient (OC) was 0.72 ± 0.02. Upon BK stimulation for 60 min, expression of both NHE-1 and ezrin was elevated in lamellipodia (Figure **2A, b**), and they remained co-localized with CC = 0.54 ± 0.04, OC = 0.74 ± 0.01 (Figure **2C** **, a**). The stimulated microglia displayed more elongated morphology, which was reflected by decreased cell roundness (Figure **2C** **, a**, 0.30 ± 0.04 vs. 0.55 ± 0.03, p < 0.05). Inhibition of NHE-1 activity with HOE 642 did not interrupt co-localization of NHE-1 and ezrin (Figures **2A, c** and **2C** **, a**), but prevented BK-mediated cell elongation. Similar results were obtained in primary microglia (Figure **2C, b**). These data imply that NHE-1 and ezrin were co-localized in microglia regardless whether they were stimulated. To further confirm the co-localization of these two proteins, we examined whether disruption of actin polymerization with Cytochalasin D (Cyt.D), a drug known to inhibit actin polymerization [24,25], would affect the co-localization of NHE-1 and ezrin. As shown in Figure **2A, d**, despite of the collapsed actin filament network reflected by cell shrinkage and retracted microglial processes (Figure **2A, d** **, inset**), condensed ezrin immunoreactive signals remained clustered with the NHE-1 immunofluorescence signals (Figure **2A, C**, CC= 0.58 ± 0.03, OC = 0.73 ± 0.02). Moreover, interactions of NHE-1 and ezrin ERM were confirmed using immunoprecipitation in BV2 cells in which NHE-1 protein was pulled down in the immunoprecipitates using the anti-ezrin antibody (Figure **2B**). In the BK-stimulated conditions, NHE-1 protein bound to ezrin ERM was further increased (2.57 ± 0.88 folds of Con, p < 0.05). Therefore, interactions of NHE-1 and ezrin may play a role in lamellipodial morphological changes and microglial movement.

### NHE-1 activity functions in H^+^ extrusion in microglial lamellipodia

**Figure 3:**
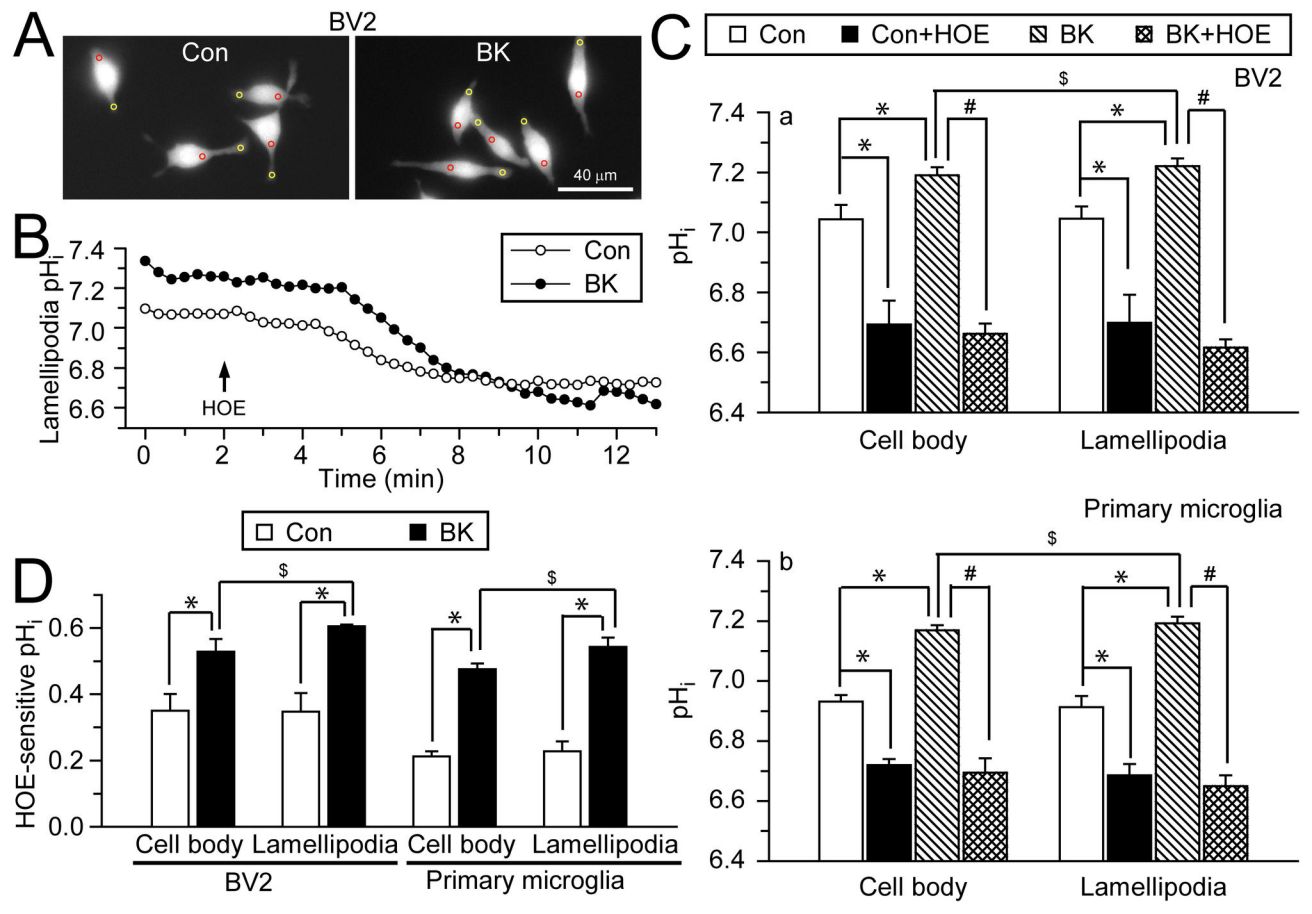
NHE-1 regulates pH_i_ of cell body and lamellipodia in microglia. **A**. BCECF-loaded images of BV2 microglia under control conditions (left panel) and after 30 min of 300 nM BK application (right panel). pH_i_ was calculated in cell body (red circles) and lamellipodia (yellow circles) of each cell. *Scale bar*: 40 µm. **B**. Representative traces of lamellipodial pH_i_ under control or BK stimulation. **C**. Summarized baseline pH_i_ and pH_i_ after 10 min of 1 µM HOE 642 in the absence or presence of BK in cell body and lamellipodia of BV2 (**a**) and primary microglia (**b**). **D**. HOE642-sensitive pH_i_ under control and BK conditions. Data are mean ± SEM (n = 3-6 independent cultures; data were calculated as an average from 8–16 cells in each culture). * p < 0.05 vs. Con. # p < 0.05 vs. BK. $ p < 0.05 vs. cell body by paired t-test.

To further understand the underlying mechanisms of NHE-1 in BK-stimulated microglial motility, we measured pH_i_ in cell body and lamellipodia of microglia with the H^+^-sensitive dye BCECF (Figure **3A**, *red and yellow circles*). BV2 microglia exhibited a basal pH_i_ of 7.04 ± 0.05 in cell body, and 7.05 ± 0.04 in lamellipodia under control conditions (in HCO_3_^-^-free HEPES/MEM solution). When NHE-1 activity was inhibited by HOE 642, the basal pH_i_ decreased to 6.69 ± 0.08 in the cell body, and 6.70 ± 0.09 in lamellipodia (Figure **3C** **, a**, p < 0.05). Similar results were observed in primary microglia (Figure 3**C**, b, p < 0.05). These data suggested that NHE-1 plays a role in regulating basal pH_i_ of microglia. But, the resting cells did not display significant differences in pH_i_ in cell body and lamellipodia.

In contrast, BK stimulation triggered an increase in pH_i_ in both lamellipodia and cell body compared to control cells (7.19 ± 0.03 in cell body, and 7.22 ± 0.03 in lamellipodia, p < 0.05). However, with BK stimulation, pH_i_ is more alkaline in lamellipodia compared to cell body (Figure **3C** **, a**, p < 0.05 by paired t-test). Interestingly, inhibition of NHE-1 with 1 µM HOE 642 acidified microglia (6.66 ± 0.03 in cell body, and 6.62 ± 0.03 in lamellipodia, p < 0.05). HOE-sensitive pH_i_ was calculated by subtracting the pH_i_ under HOE 642-treatment from baseline pH_i_ in both control and BK-stimulated condition (Figure **3D**). These data suggest that NHE-1 is important in maintaining alkaline pH_i_ after BK stimulation, and NHE-1 activity is elevated by BK.

1 µM HOE 642 is an optimal dose to selectively block NHE-1. We performed dose–response experiments with HOE 642 (Figure **S3**). We measured the changes in resting pH_i_ in BV2 microglia after treatment with 4 different concentrations of HOE 642: 0.1, 0.5, 1, or 10 µM. As shown in Figure **S3**, 0.1 µM of HOE 642 did not cause significant change in pH_i_. HOE 642 acidified microglia in dose-dependent fashion from 0.5–1 µM. 10 µM of HOE 642 did not further acidify microglia. HOE 642 has an IC_50_ of 0.03-3.4 µM on NHE-1 and 4.3-62 µM on NHE-2 [26]. The lack of differences between effects of 1 and 10 µM HOE 642 on microglial pH_i_ suggests that NHE-1 is a dominant isoform in microglia.

### Coupled activation of NHE-1 and reverse mode operation of Na^+^/Ca^2+^ exchange (NCX_rev_) leads to intracellular Ca^2+^ elevation in BK-stimulated microglia

**Figure 4:**
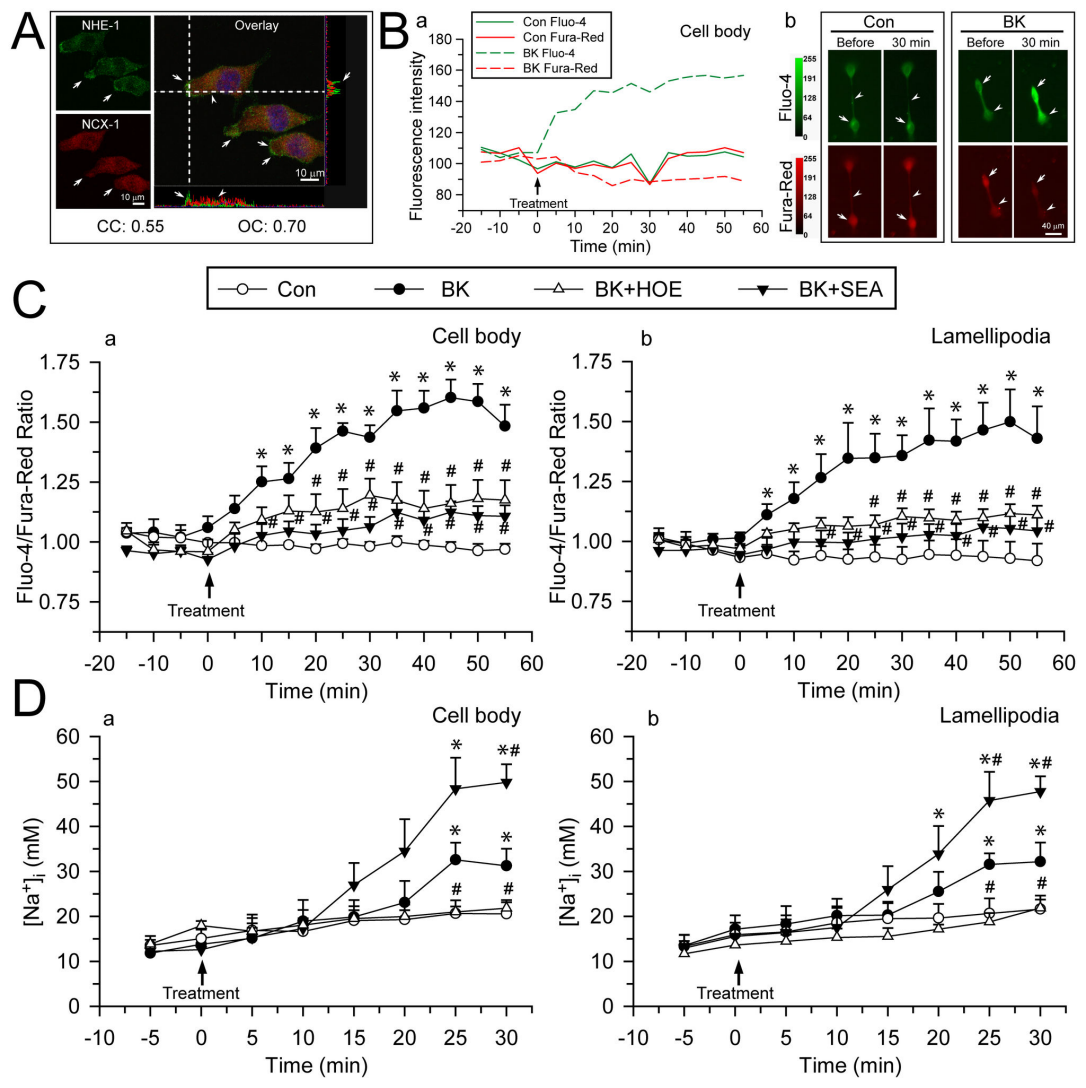
BK-mediated elevation of intracellular Ca^2+^ and Na^+^ results from concurrent activation of NHE-1 and NCX_rev_. **A**. Double immunostaining of NHE-1 (Green) and NCX-1 (Red) in BV2 microglia under control conditions. *Blue*: To-pro-3 nuclear staining. Co-localization of green and red signal and their intensity spectrum were shown along the white dashed lines on the overlay images. *Arrow*: Higher expression level of NHE-1 than NCX-1 in lamellipodia edge. *Arrowhead*: Colocalization of NHE-1 and NCX-1 in cell body. *Scale bar*: 10 µm. Pearson’s correlation coefficient (CC) and overlap coefficient (OC) values for NHE-1 and NCX-1 signals were shown. **B**. (**a**) Representative fluorescence intensity changes of Fluo-4 (Green) and Fura-Red (Red) in BV2 cell body. (**b**) Representative fluorescence images of BV2 microglia in the absence or presence of BK (300 nM) for 0-30 min. *Arrow*: cell body; *arrowhead*: lamellipodia. *Scale bar*: 40 µm. **C**. Summarized data of BK-induced Ca^2+^_i_ elevation in BV2 cell body (**a**) and lamellipodia (**b**). For HOE and SEA treatment, 1 µM HOE 642 or 1 µM SEA 0400 was used. Data are mean ± SEM (n = 4-6 independent cultures, data were calculated from 12-19 cells for each group). **D**. Summarized changes of [Na^+^]_i_ in primary microglia under control, BK, HOE 642 or SEA 0400 treatment in cell body (**a**) and lamellipodia (**b**). Data are mean ± SEM (n = 5-7 independent cultures, data were calculated from 15-21 cells for each group). * p < 0.05 vs. corresponding Con; # p < 0.05 vs. corresponding BK.

It has been reported that NCX_rev_ plays a key role in Ca^2+^ influx in migrating microglia [7]. However, it remains unknown what causes NCX_rev_ activation in microglial cells. We speculated that NHE-1-mediated Na^+^ overload may be in part responsible for NCX_rev_ activation. First, NHE-1 and NCX-1 immunoreactive signals were largely colocalized in cell body as well as in lamellipodia, as reflected by the colocalization coefficients (CC = 0.55, OC = 0.70). Interestingly, NHE-1 had higher expression levels than NCX-1 at lamellipodia edge (Figure **4A**, **arrows**). We then determined whether BK stimulation led to NCX_rev_ activation and Ca^2+^ influx. The emission ratio of Fluo-4/Fura-Red, was used to determine changes of Ca^2+^_i_ because it is independent of changes in cell volume [27]. Ca^2+^ concentration in BV2 cells was not changed under control conditions. Upon exposure to BK, BV2 cells exhibited an increase in Fluo-4 whereas a slow decrease in Fura-Red fluorescence signals (Figure **4B**). Figure **4C** further illustrated time-dependent changes of the Fluo-4/Fura-Red ratios in cell body and lamellipodia of BV2 cells. The baseline of Fluo-4/Fura-Red ratio in control BV2 cell body and lamellipodia was 1.04 ± 0.02 and 1.02 ± 0.01, respectively. BK induced a significant rise in Ca^2+^_i_, with an increase in Fluo-4/Fura-Red ratio of 1.25 ± 0.06 fold in cell body at 10 min of BK treatment or a 1.18 ± 0.07 fold increase in lamellipodia at 10 min BK treatment (p < 0.05, Figure **4C**). The Fluo-4/Fura-Red ratio reached the peak at 50-55 min of BK treatment in cell body and lamellipodia. Interestingly, specific NCX_rev_ inhibitor SEA 0400 (1µM) nearly abolished the BK-induced rise in Ca^2+^_i_ in BV2 cells (p < 0.05, Figure **4C**). Moreover, inhibition of NHE-1 with NHE inhibitor HOE 642 (1µM) also prevented BK-mediated Ca^2+^_i_ rise (Figure **4C**). A similar pattern of Ca^2+^_i_ rise was observed in primary microglia (Figure **S4**). These results clearly imply that NHE-1 activity plays a role in regulation of NCX_rev_ and BK-induced Ca^2+^_i_ elevation upon BK stimulation.

SEA0400 is a specific NCX inhibitor and has been proved to strongly inhibit NCX activity in cultured cells (rat neurons, astrocytes and microglia) with IC_50_ values of 5–33 nM [28]. To further validate the optimal dose of SEA0400 at 1 µM, we performed additional dose–response experiments with SEA0400 (0.1, 1, 10µM). As shown in Figure **S5**, BK-induced intracellular Ca^2+^ elevation was significantly blocked at 1 µM of SEA04000. There was no notable difference between effects of 1 µM and 10 µM SEA0400. In contrast, 0.1 µM SEA0400 was partially effective.

### NHE-1 activation causes intracellular Na^+^ overload in BK-stimulated microglia

We speculated that NHE-1 involves in NCX_rev_-mediated Ca^2+^ signaling via Na^+^_i_ overload in the BK-stimulated microglia. As shown in Figure **4D**, basal level of Na^+^_i_ was 15.1 ± 2.0 mM in primary microglial cell body. Upon BK stimulation (25 min), Na^+^_i_ increased to 32.6 ± 3.8 mM in cell body and 31.5 ± 2.5 mM in lamellipodia. In contrast, in the presence of NHE-1 inhibitor HOE 642, microglia failed to show significant increases in Na^+^_i_ in cell body (21.8 ± 1.3 mM) or in lamellipodia (21.8 ± 1.9 mM) after 30 min BK treatment. Most interestingly, inhibition of NCX_rev_ with its inhibitor SEA0400 (1µM) caused further Na^+^_i_ overload in cell body (49.8 ± 4.0 mM) and in lamellipodia (47.8 ± 3.4 mM) after 30 min BK treatment (p < 0.05, Figure **4D**). These data strongly suggest that NCX functions in the reverse mode in extrusion of Na^+^ with exchange of Ca^2+^ influx. Therefore, coupling NHE-1 activation with reversal of NCX function maintains not only intracellular H^+^ but also Na^+^ homeostasis in microglia.

### NHE-1 is involved in BK-induced microglial motility

**Figure 5:**
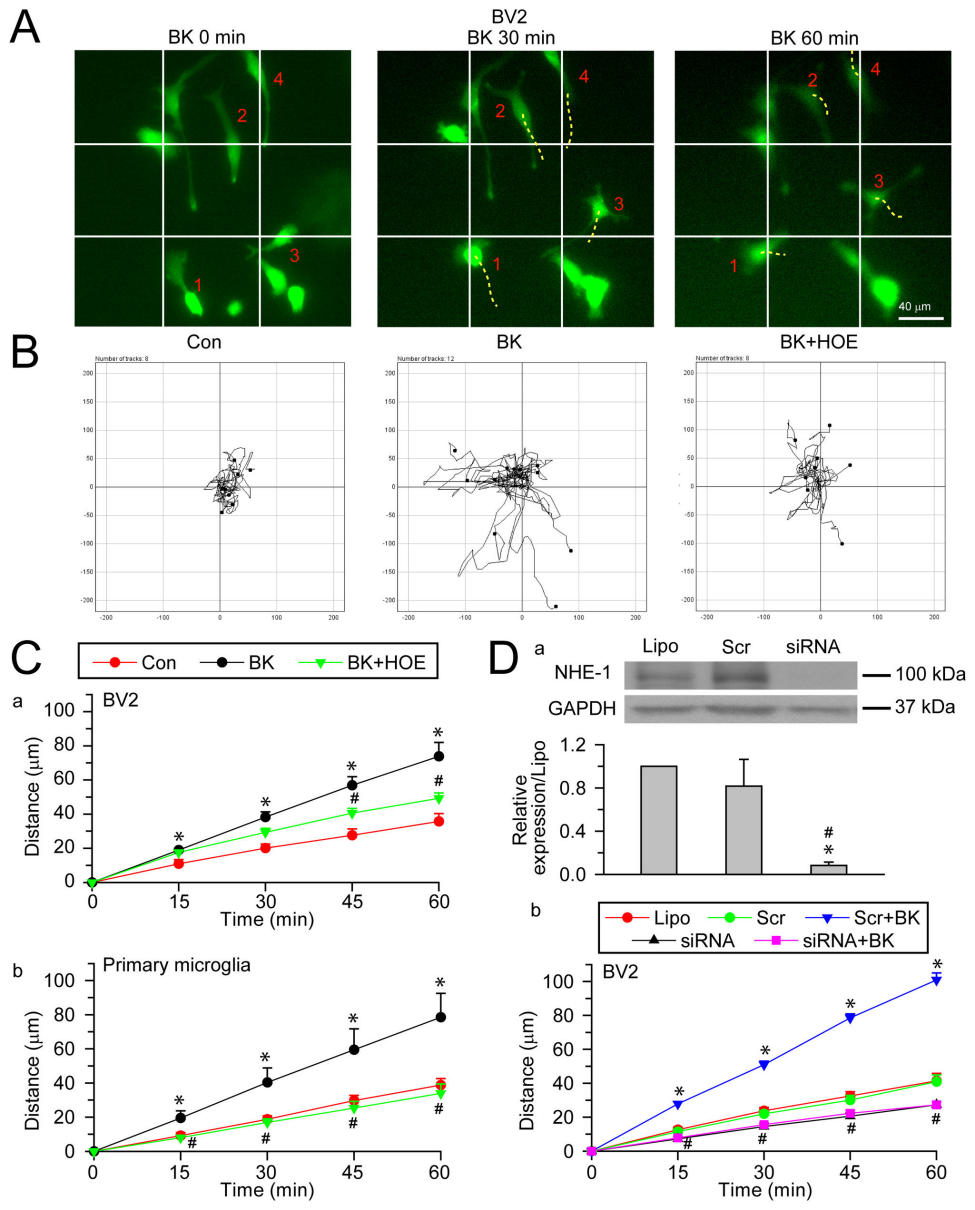
Inhibition of NHE-1 activity abolishes BK-stimulated microglial motility. **A**. Representative images of calcein AM loaded BV2 in the presence of 300 nM BK for 0-60 min. Cells were loaded with calcein AM (0.5 µM) for 30 min prior to imaging. Moving cells (4) in the field were marked (#1-4). *Solid line*: reference. *Dashed line*: changes in cell gravity center compared to the previous image. *Scale bar*: 40 µm. **B**. Motility of BV2 or primary microglia was recorded using the Nikon TiE time-lapse imaging system. Cells were exposed to 300 nM BK in the absence or presence of 1 µM HOE 642 for 60 min. Random moving traces of 8-12 representative cells under control, BK and BK plus HOE conditions in the 60-min recording period were shown. **C**. Summary data of BK-mediated microglial motility. Accumulated distance of BV2 (**a**) or primary microglia (**b**) cell movement during 0-60 min was calculated in unstimulated (Con), BK-treated (BK), or cells treated with BK and HOE 642 (BK + HOE). Data are mean ± SEM (n = 4). * p < 0.05 vs. Con. # p < 0.05 vs. BK. **D**. Effects of NHE-1 siRNA on NHE-1 expression and BV2 cell motility. **a**. Representative blots of NHE-1 protein expression (upper panel) and summarized data as NHE-1 expression normalized with GAPDH (lower panel). Lipo: Lipofectamine, Scr: control scramble siRNA or siRNA: NHE-1 siRNA. Data are mean ± SEM. n = 3. * p < 0.05 vs. Lipo. # p < 0.05 vs. Scr. **b**. BV2 cell motility. Data are mean ± SEM. n = 3-4. * p < 0.05 vs. Scr. # p < 0.05 siRNA + BK vs. Scr + BK.

We then investigated whether increased NHE-1 activity in lamellipodia contributes to microglial random cell movement. Images of BV2 cells in Figure **5A** illustrated individual cell positions prior to, at 30 or 60 min after exposure to BK. Many cells displayed position changes during 30-60 min exposure to BK (Figure **5A**, dashed lines of cell #1-4). In addition, Figure **5B** illustrated the random moving traces of 8-12 representative cells under control conditions, showing little motility. In the presence of BK, the microglial motility was clearly increased. In contrast, this increased random movement of microglia was inhibited by inhibition of NHE-1 with HOE 642 (Figure **5B**). Summarized data were shown in Figure **5C**. The unstimulated BV2 cells exhibited minimum movement over 60 min. In the presence of BK, BV2 motility was significantly increased during 60 min, from 35.7 ± 4.6 µm to 73.8 ± 8.2 µm (p < 0.05). Similar results were obtained in primary microglia where BK induced a 102% increase in total moving distances at 60 min (78.5 ± 24.4 µm vs. 38.8 ± 6.7 µm, n = 4, p < 0.05, Figure **5C, b**). BK increased the average moving speed of BV2 cells by 106% (1.2 ± 0.1 µm/min vs. 0.60 ± 0.08 µm/min, p < 0.05) and 102% in primary microglia (1.31 ± 0.41 µm/min vs. 0.65 ± 0.11 µm/min, p < 0.05). In comparison, HOE 642-treated BV2 cells showed significantly reduced motility at 60 min (49.23 ± 3.18 µm in BV2 and 33.93 ± 9.58 µm in primary microglia, p < 0.05, Figure **5C**). HOE 642 also reduced microglial moving speed by 33% in BV2 (0.82 ± 0.05 µm/min, p < 0.05) and 56% in primary microglia (0.57 ± 0.16 µm/min, p < 0.05). In Summary, BK increased microglial motility and inhibition of NHE-1 activity abolished the BK-induced stimulation in cell motility.

To further validate that BK+HOE 642 treatment does not cause cytotoxicity in microglia, healthy morphology of microglia and well-retained calcein dye (more than 96% cells) in the BK+HOE 642-treated microglia were shown in Figure **S6**. This illustrates the well-preserved plasma membrane integrity of microglia under these conditions.

We further investigated the function of NHE-1 in microglial movement by knocking down the NHE-1 protein expression in BV2 cells with siRNA. As shown in Figure **5D**, control scramble siRNA (Scr) did not cause significant change in NHE-1 expression compared to cells transfected with Lipofectamine (Lipo) (81.5 ± 24.8%, normalized with GAPDH expression, p = 0.50), but NHE-1 siRNA knocked down ~92% of NHE-1 protein. We then monitored the total moving distances of these cells over 0-60 min BK stimulation. Scr siRNA-transfected cells showed no significant change in motility. BK induced increased cell motility in Scr siRNA-treated cells (100.9 ± 4.2 µm vs. 40.9 ± 4.0 µm, n = 3-4, p < 0.05). Interestingly, NHE-1 siRNA-treated cells exhibited significantly reduced basal cell motility (Figure 5**D**, b, p < 0.05) and failed to show BK-mediated stimulation in motility (Figure **5D**). Moreover, siRNA knockdown of NHE-1 also reduced BK-mediated changes in lamellipodial dynamics, in a similar manner as HOE 642 treatment. The lamellipodial area was reduced to 198.9 ± 44.7 µm^2^ and the persistence rate was increased to 19.1 ± 2.2 min (n = 3, data not shown). Taken together, these data imply that NHE-1 activity and its structural interactions with other proteins are required in BK-induced microglial motility.

### Inhibition of NHE-1 activity abolishes BK-induced microglial migration

**Figure 6:**
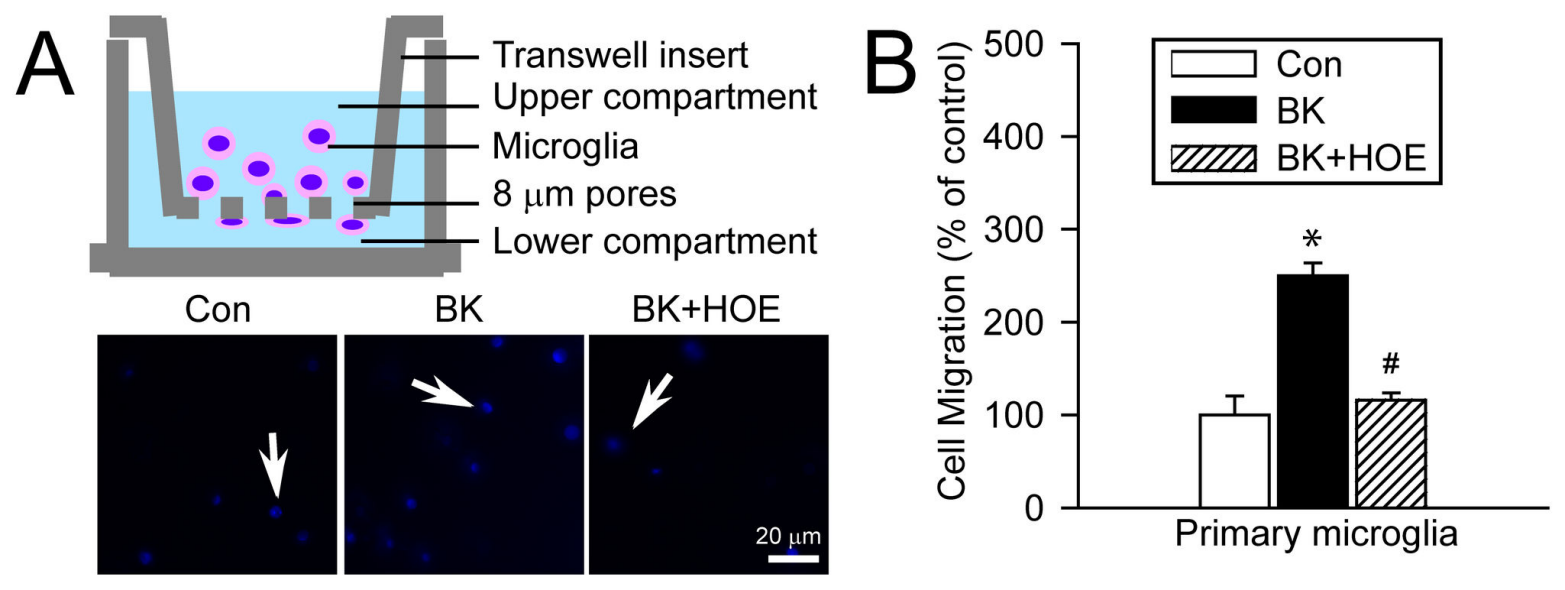
BK-induced microglial microchemotaxis depends on NHE-1 activity. **A**. BK-induced microchemotaxis of primary microglia using the Boyden Chamber (8 µm pore) for 5 h. To inhibit NHE-1 activity, 1 µM HOE 642 was added into the upper compartment. *Blue*: DAPI stained cells. *Scale bar*: 20 µm. **B**. Summary data. Data are mean ± SEM (n = 3 independent cultures). * p < 0.05 vs. Con. # p < 0.05 vs. BK.

We further investigated primary microglia migration with transwell chemotaxis in the Boyden chamber and migrated cells were stained with DAPI (Figure **6A** **, arrow**). Figure **6B** shows that BK triggered a 250 ± 13.9% increase in trans-well migration of primary microglia (p < 0.05). In contrast, in the presence of HOE 642, BK-mediated chemotaxis was reduced to 116 ± 7.8% of the control. Taken together, these results allow us to firmly conclude that NHE-1 activity is involved in the BK-induced transwell chemotaxis of microglia.

### BK-induced microglial motility depends on intracellular Ca^2+^ signaling

**Figure 7:**
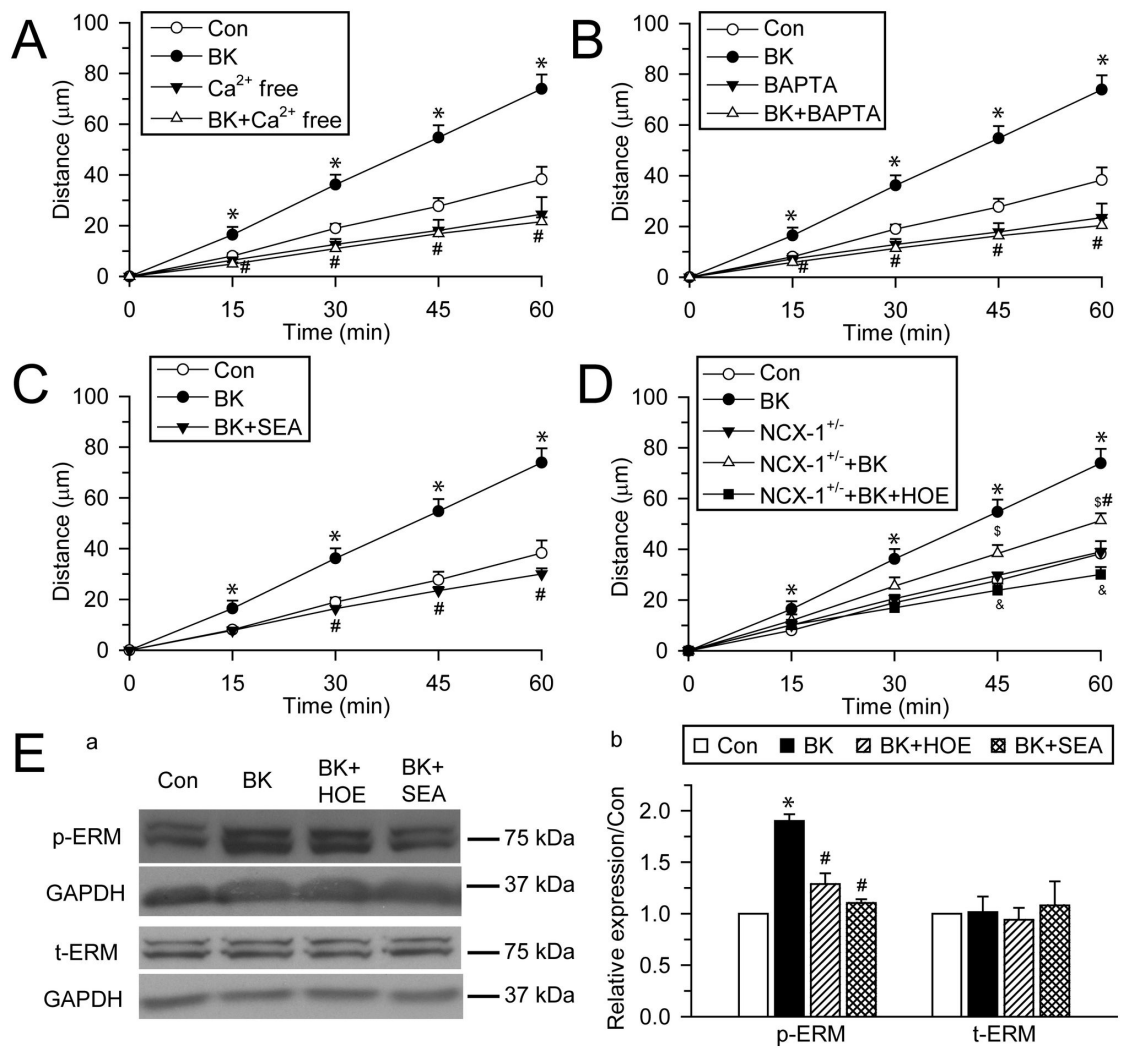
BK-induced microglial motility depends on function of NHE-1 and NCX_rev_. **A**. Removal of extracellular Ca^2+^ abolishes BK-mediated stimulation of primary microglia motility. Random movement of primary microglia was monitored for 60 min and total accumulated moving distance was summarized. **B**. Intracellular Ca^2+^ chelator BAPTA AM (10 µM) abolishes microglial motility. **C**. BK-induced microglial motility was inhibited by NCX_rev_ activity inhibitor SEA 0400 (1 µM). **D**. Less microglial motility in NCX-1^+/-^ microglia. NHE-1 inhibitor HOE 642 (1µM) completely abolished NCX-1^+/-^ primary microglia motility induced by BK. Data are mean ± SEM (n = 3-5 independent cultures, data were calculated from 12-20 cells for each group). * p < 0.05 vs. Con. # p < 0.05 vs. BK. $ p < 0.05 vs. NCX-1^+/-^. & p < 0.05 vs. NCX-1^+/-^ + BK. **E**. BV2 cells were stimulated with 300 nM BK or 1 µM HOE 642 or SEA 0400 for 15 min and cell lysate was collected for immunoblotting analysis. **a**. Representative blots of phosphorylated ERM (p-ERM) and total ERM (t-ERM) expression. GAPDH was used as internal control. **b**. Summarized data of p-ERM and t-ERM expression normalized with GAPDH level. Data were calculated as relative expression to Con and expressed as mean ± SEM. n = 3-4. * p < 0.05 vs. Con. # p < 0.05 vs. BK.

We hypothesized that NHE-1 affects microglial migration in part via triggering NCX_rev_-mediated Ca^2+^ signaling. First, we determined whether microglial migration depends on Ca^2+^_i_. As shown in Figure 7A, B, either removal of extracellular Ca^2+^ (Ca^2+^ free extracellular solution) or chelating Ca^2+^_i_ with intracellular Ca^2+^ chelator BAPTA AM (10 µM) abolished BK-induced increase in microglia motility. These results indicate that Ca^2+^ influx and the subsequent increase in Ca^2+^_i_ are necessary for the BK-induced microglial mobility.

To examine whether Ca^2+^ influx via NCX_rev_ is involved in BK-induced microglial motility, we used the NCX_rev_ inhibitor SEA 0400 as well as transgenic heterozygous Na^+^/Ca^2+^ exchanger isoform 1 primary microglia (NCX-1^+/-^). In the presence of SEA 0400 (1 µM), BK-induced increase in microglial motility was completely blocked (Figure **7C**). In contrast, NCX-1^+/-^ microglia showed less BK-induced stimulation of cell motility (Figure **7D**). Moreover, inhibition of NHE-1 with HOE 642 (1µM) abolished the BK-induced remaining motility in NCX-1^+/-^ microglia (Figure **7D**). Taken together, these results strongly suggest that NCX_rev_ activation play a role in the BK-induced microglial motility. More importantly, NHE-1 activation functions as an upstream regulator of NCX_rev_ activation.

We speculated that NHE-1 and NCX_rev_ regulate microglial motility in part via stimulating ERM phosphorylation and activation. Phosphorylation of ERM at the C-terminal threonine residue depends on Ca^2+^_i_ and plays a role in modulating the conformation and function of ERM proteins [29,30]. As shown in Figure **7E**, 15 min of BK stimulation induced phosphorylation of ERM (190 ± 6% of control, p < 0.05), while the expression of total ERM protein remained unchanged (101 ± 15%, p = 0.24). Interestingly, BK-mediated effects on ERM phosphorylation were significantly reduced by NHE-1 inhibition (128 ± 10% of control, p < 0.05). SEA 0400 also reduced BK-induced ERM phosphorylation (Figure **7E**, 110 ± 3% of control vs. 190 ± 6%, p < 0.05). These data strongly suggest that NHE-1 regulates microglial motility in part via facilitating NCX_rev_-mediated Ca^2+^ signaling and ERM phosphorylation.

## Discussion

### NHE-1 in microglial migration

Migration is an important component of microglial activation in response to various pathological stimuli. Despite that NHE-1 has been shown to be involved in migration of smooth muscle and fibroblast cells [14,31], it remains unknown whether NHE-1 regulates microglial migration. In this study, we first detected that microglial lamellipodia at the leading edge displayed highly motile dynamics upon BK stimulation. A more dynamic lamellipodia is a driving force for increased cell motility in macrophages [32]. Interestingly, inhibition of NHE-1 with its potent inhibitor HOE 642 blocked these changes. Moreover, BK induced the accumulation of F-actin in lamellipodia, which is required in the membrane protrusion formation at leading edge during cell movement [33]. We found that blocking NHE-1 activity with HOE 642 inhibited the F-actin accumulation in lamellipodia. Most importantly, either inhibition of NHE-1 activity with HOE 642 or knockdown of NHE-1 expression with siRNA significantly reduced BK-mediated microglial random movement. These data provide the first line of evidence that NHE-1 plays an important role in microglial migration.

### NHE-1 regulates microglial migration via maintaining pH_i_

NHE-1 catalyzes the secondary electroneutral transport of Na^+^ influx in exchange of H^+^ extrusion and is a main mechanism in regulating pH_i_ in microglia [17]. In the present study, we observed an increase in pH_i_ in the HCO_3_^-^-free buffer upon BK stimulation, especially in lamellipodia. Moreover, the BK-treated microglia were more sensitive to NHE-1 inhibitor HOE 642-mediated inhibition of H^+^ extrusion, suggesting that the BK-induced increase in pH_i_ largely results from stimulating NHE-1 activity.

Maintaining alkaline pH_i_ by NHE-1 is important in microglial migration. pH_i_ serves as a regulator of cell polarization and migration [34]. pH_i_ gradients have been detected in migrating melanoma and fibroblast cells, with more alkaline pH_i_ at leading edge and decreased pH_i_ in cell body and further the rear edge [35]. Fibroblast lacking the ion translocation function of NHE-1 protein exhibits impaired motility [14]. However, how the local pH_i_ environment regulates cell movement is not fully understood and it has been linked to regulation of the actin binding protein cofilin activity. Activation of cofilin promotes accumulation of actin at leading edge and is required for cell motility [33,36]. Cofilin activity was inhibited by pH-dependent phosphoinositide binding at more acidic pH_i_ [13]. In contrast, more alkaline pH_i_ environment increases activity of cofilin to promote actin filament dynamics [37]. Therefore, NHE-1 may regulate microglial migration in part by providing an optimal local alkaline pH_i_ environment.

### Interactions between NHE-1 and cytoskeletal proteins in microglia

NHE-1 can also regulate microglial structure via direct interactions with cytoskeletal proteins. The association of actin filaments with the plasma membrane provides mechanical stability, maintains cell shape and adhesion, and regulates cell movement [11]. The link between the actin cytoskeleton and the plasma membrane is facilitated by a group of actin-binding proteins, which includes the ERM proteins ezrin, radixin, and moesin [22,23]. A number of integral membrane proteins anchor actin filaments and the cortical cytoskeletal network to the plasma membrane, including adhesion molecules and ion transporters. In fibroblasts, NHE-1 is predominantly localized in lamellipodia, where it functions as a plasma membrane anchor for actin filaments by its direct binding of ERM [9]. Moreover, lacking NHE-1 in *Dictyostelium* cells led to impaired chemotaxis and the assembly of F-actin [21].

In the current study, we detected a co-localization of NHE-1 and ezrin in migrating microglia. Under both control and BK-stimulated conditions, the Pearson’s coefficient and overlap coefficient for NHE-1 and ezrin showed positive correlation. The interactions between NHE-1 and ezrin were further validated in Cyt.D study as well as in immunoprecipitation study. Despite of the Cyt.D-mediated devoid of actin filaments in microglia, NHE-1 and ezrin remained co-localized. These data clearly illustrate that NHE-1 interacts with ERM, which can promote actin filament dynamics and microglial migration.

### Coupling of NHE-1 activation with NCX_rev_ is important for BK-induced microglia motility

BK stimulates microglial motility through activation of BK receptor-mediated signaling pathways [7,38], which includes synthesis of diacylglycerol (DAG) and activation of PKC [7]. In particular, an early transient increase in Ca^2+^_i_ via a Ca^2+^ influx is important in microglial migration stimulated by BK [7,39]. In the present study, either removal of extracellular Ca^2+^, or chelating Ca^2+^_i_ inhibited the BK-induced microglial migration. Moreover, BK stimulated ERM phosphorylation. Either blocking NHE-1 or NCX_rev_ abolished the BK-mediated ERM stimulation. These data suggest that NCX_rev_-mediated Ca^2+^ rise is a key signaling molecule in the BK-induced microglial motility.

How NCX_rev_ is regulated in microglia is not well understood. Ifuku et al. reported that BK-mediated PKC activation may stimulate NCX_rev_ through protein phosphorylation in microglia [7]. Interestingly, we detected a close relationship between activation of NHE-1 and NCX_rev_. We speculate that NHE-1-mediated Na^+^_i_ overload triggers activation of NCX_rev_-mediated Ca^2+^ rise. This view is supported by the following findings: 1) colocalization of NHE-1 and NCX-1 in microglia; 2) inhibition of NCX_rev_ with SEA 0400 further elevated Na^+^_i_ overload, suggesting the reverse mode operation of NCX, 3) inhibition of NHE-1 abolished the NCX_rev_-mediated Ca^2+^ rise, 4) BK induced Na^+^_i_ rise and it was blocked by NHE-1 inhibition. Thermodynamic analysis reveals that when Na_i_ increases from a basal level of 15 mM to 23 mM in oligodendrocytes, it could trigger NCX_rev_ in the absence of membrane depolarization [40]. Therefore, this mechanism may also function in regulation of NCX_rev_ in microglia in response to BK stimulation. Moreover, similar results were obtained from NCX-1^+/-^ microglia and NCX-1^+/+^ microglia treated with SEA0400. NCX-1 protein was decreased in neurons (40%) and in astrocytes (70%) or in brain tissues of NCX-1^+/-^ mouse [16,41]. We speculate that there is a significant reduction of NCX-1 protein expression in NCX1^+/-^ microglia.

We found the microglial Ca^2+^_i_ elevation preceded the Na^+^_i_ rise in response to BK treatment. The possible explanation for this phenomenon is that cells can maintain Na^+^_i_ homeostasis better than Ca^2+^_i_, mainly through Na^+^–K^+^-ATPase and NCX_rev_. This speculation was supported by our findings that when Na^+^–K^+^-ATPase was partially inhibited by ouabain, Na^+^_i_ was elevated faster in the BK-treated microglia than in the control microglia (Figure **S7**). Taken together, our data strongly suggest that NHE-1 activation in microglia is functionally coupled with NCX_rev_ in response to BK. The concerted activation of NHE-1 and NCX_rev_ is important for NCX_rev_-mediated Ca^2+^ influx and microglia motility.

In this study, we have only characterized function of NHE-1 in regulation of microglia migration in cultures and under conditions in response to the stimulus BK. Additional studies are needed to validate these mechanisms in *in vivo* models as well as using other stimuli which selectively trigger either “classical” or “alternative” polarized activation of microglia.

## Conclusions

**Figure 8:**
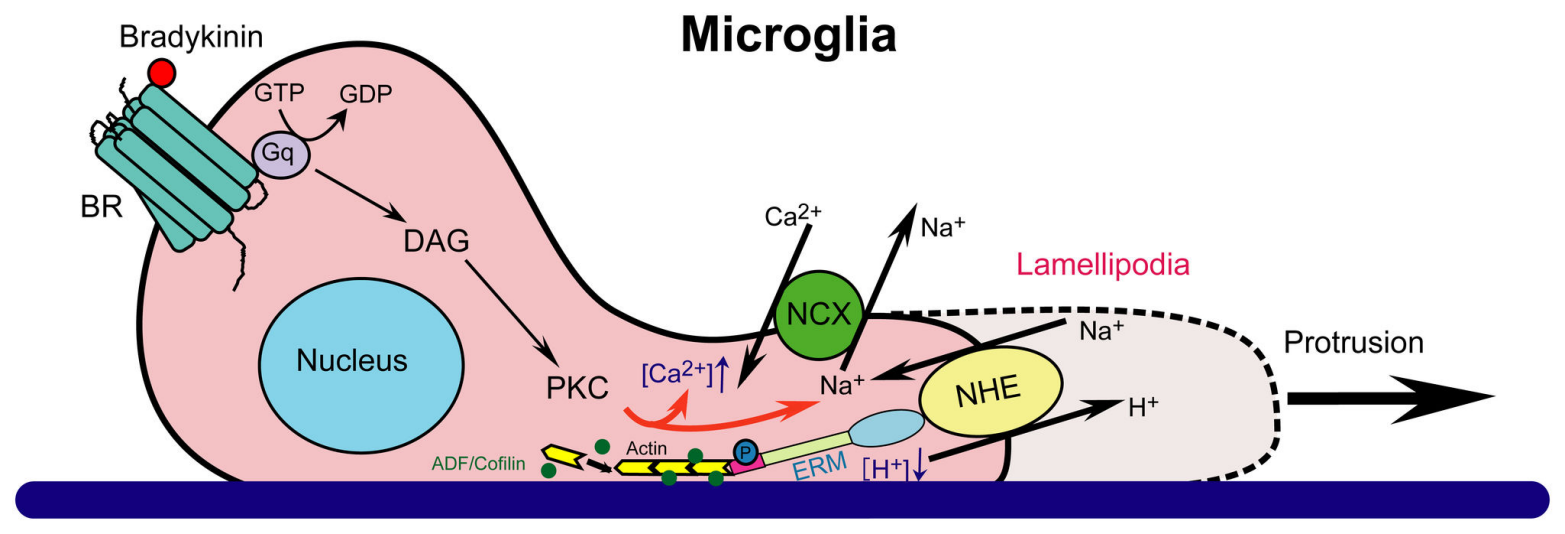
Proposed mechanisms underlying NHE-1-mediated regulation of microglial migration. NHE-1 interacts with ERM proteins and functions as an anchoring point for actin filament, contributing to membrane protrusion and microglial movement. BK, a chemoattractant produced in injured brain tissues, can induce microglial migration. BK activates the G-protein coupled BK receptor and results in the downstream signaling cascade including activation of phospholipase C (PLC), synthesis of inositol triphosphate (IP_3_) and diacylglycerol (DAG), and activation of protein kinase C (PKC). BK stimulates microglial NHE-1 activity to maintain an alkaline pH_i_ in lamellipodia, which facilitates the pH_i_-sensitive actin binding proteins ADF/cofilin function during microglial movement. In addition, NHE-1 mediated Na^+^ influx triggers the NCX_rev_ operation and Ca^2+^_i_ rise, which further facilities ERM activation and actin accumulation. Taken together, NHE-1 plays an important role in microglial migration.

In summary (Figure **8**), we report here that BK-mediated stimulation of NHE-1 activity is important in microglial migration. NHE-1 functions to maintain an alkaline pH_i_, especially in lamellipodia, which is critical for actin accumulation and microglia migration. NHE-1 protein also interacts with ERM protein and provides anchoring for actin cytoskeleton. Moreover, NHE-1-mediated Na^+^_i_ overload triggers reversal of NCX function and Ca^2+^_i_ rise and further facilitates phosphorylation of ERM and microglial migration.

## Supporting Information

Figure S1 **siRNA specifically downregulates NHE-1 protein expression level in BV2 microglia.** BV2 cells were transfected with Lipofectamine only (Lipo), control scramble siRNA (Scr) or two NHE-1 siRNAs with different sequences (siRNA1: forward, 5’-CCACAAUUUGACCAACUUAtt-3’; reverse, 5’-UAAGUUGGUCAAAUUGUGGtc-3’. siRNA2: forward, 5’-CGAAGAGAUCCACACACAGtt-3’; reverse, 5’-CUGUGUGUGGAUCUCUUCGtt-3’). Representative immunoblots of NHE-1, NCX-1 and tERM protein expression were shown. Expression of GAPDH in each blot was shown as internal control for protein loading. Expected protein size: NHE-1: 90-110 kDa; NCX-1: 120 kDa (full length protein), 160 kDa (non-reduced protein even in the presence of DDT) and 70 kDa (active proteolytic fragment); tERM: 75 kDa (moesin) and 80 kDa (ezrin and radixin).
(TIF)

Figure S2 **NHE-1 is involved in basal movement of microglia.** BV2 microglial movement was monitored for 60 min in the presence or absence of 1 µM HOE 642. **A**. Summarized lamellipodial area (**a**), persistence (**b**), protrusion rate (**c**) and area change (**d**) were shown. **B**. Accumulated moving distance during 0-60 min was shown. Data were mean ± SEM. n =3 independent cultures. * p < 0.05 vs. Con.
(TIF)

Figure S3 **Inhibition of NHE-1 with HOE-642 abolishes pH_i_ regulation in BV2 microglia in a dose-dependent manner.** BV2 cells were loaded with 1.5 µM BCECF and monitored for 14 min. HOE 642 was added at 2 min. Traces of BV2 cell body pH_i_ in response to 0, 0.1, 0.5, 1, or 10 µM of HOE 642 were shown.
(TIF)

Figure S4 **BK-mediated elevation of intracellular Ca^2+^ in primary microglia depends on concurrent activation of NHE-1 and NCX_rev_.** Summarized data of BK-induced intracellular Ca^2+^ elevation in primary microglial cell body (**left panel**) and lamellipodia (**right panel**) were shown. 300 nM BK was used to induce intracellular Ca^2+^ elevation. For HOE and SEA treatment, 1 µM HOE 642 or 1 µM SEA 0400 was given together with 300 nM BK. Inhibition of NHE-1 or NCX_rev_ activity with HOE 642 or SEA 0400 abolished Ca^2+^_i_ elevation induced by BK. Data were mean ± SEM (n = 3 independent cultures, data were calculated from 9–12 cells for each group). * p < 0.05 vs. corresponding Con; # p < 0.05 vs. corresponding BK.
(TIF)

Figure S5 **NCX_rev_ functions in BK-mediated elevation of intracellular Ca^2+^ in BV2 cells.** Summarized data of SEA0400 dose-dependent blockade of Ca^2+^_i_ elevation in BV2 cells were shown. For SEA treatment, 0.1, 1 or 10 µM SEA 0400 was used. Data were mean ± SEM (n = 4-6 independent cultures, data were calculated from 12-19 cells for each group). * p < 0.05 vs. corresponding Con; # p < 0.05 vs. corresponding BK.
(TIF)

Figure S6 **Evaluation of BV2 microglial viability during time-lapse motility monitoring.** BV2 microglia were loaded with calcein AM (0.5 µM) for 30 min prior to the 60-min time-lapse imaging as described in Methods. **A**. Representative images of BV2 cells under brightfield (BF) or calcein AM loaded BV2 cells after 60 min of treatment were shown: control (Con), 1 µM HOE 642 (Con+HOE), 300 nM BK (BK), or 300 nM BK + 1 µM HOE 642 (BK+HOE). *Scale bar*: 20 µm. **B**. Calcein-poistive cells were counted in each group and normalized with total cells shown under brightfield. Data were mean ± SEM. n = 2 independent cultures.
(TIF)

Figure S7 **Inhibition of Na^+^–K^+^-ATPase function unmasks intracellular Na^+^ overload in microglia.** Summarized data of [Na^+^]_i_ in primary microglial cell body were shown. 0.5 mM ouabain was added to inhibit Na^+^-K^+^-ATPase activity in the absence or the presence of 300 nM BK. BK induced a faster increase in [Na^+^]_i_ in the presence of ouabain. Data are mean ± SEM (n = 4 independent cultures, data were calculated from 12-16 cells for each group). * p < 0.05 vs. corresponding Con.
(TIF)
