# Supplementary figures and images for: Stimulation of Na+/H+ Exchanger Isoform 1 Promotes Microglial Migration
Source: PLoS One. 2013 Aug 21;8(8):e74201. doi: 10.1371/journal.pone.0074201 (PMC3749130; doi:10.1371/journal.pone.0074201)

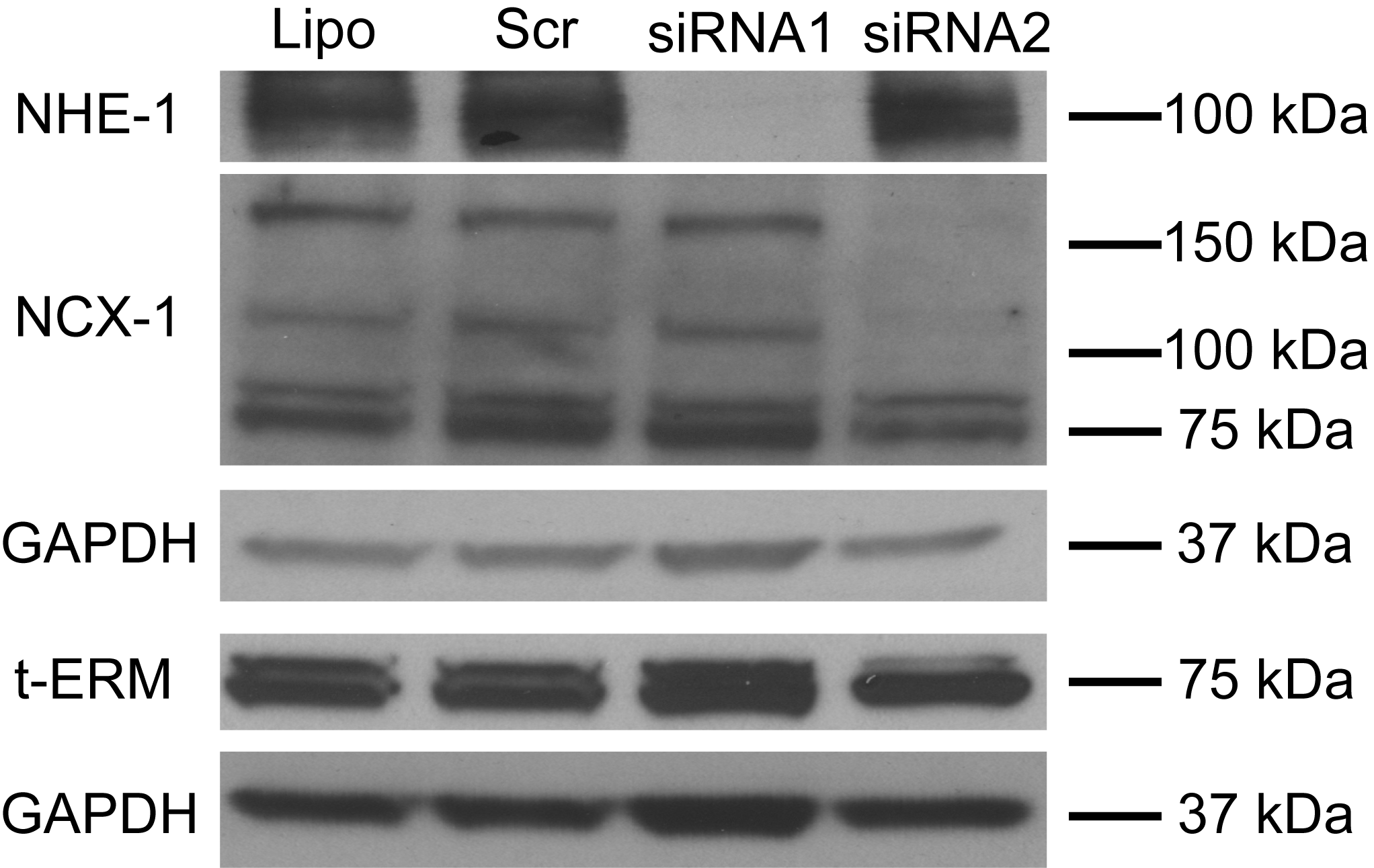

Supplement: Figure S1 — siRNA specifically downregulates NHE-1 protein expression level in BV2 microglia. BV2 cells were transfected with Lipofectamine only (Lipo), control scramble siRNA (Scr) or two NHE-1 siRNAs with different sequences (siRNA1: forward, 5’-CCACAAUUUGACCAACUUAtt-3’; reverse, 5’-UAAGUUGGUCAAAUUGUGGtc-3’. siRNA2: forward, 5’-CGAAGAGAUCCACACACAGtt-3’; reverse, 5’-CUGUGUGUGGAUCUCUUCGtt-3’). Representative immunoblots of NHE-1, NCX-1 and tERM protein expression were shown. Expression of GAPDH in each blot was shown as internal control for protein loading. Expected protein size: NHE-1: 90-110 kDa; NCX-1: 120 kDa (full length protein), 160 kDa (non-reduced protein even in the presence of DDT) and 70 kDa (active proteolytic fragment); tERM: 75 kDa (moesin) and 80 kDa (ezrin and radixin). (TIF) [file pone.0074201.s001.tif]

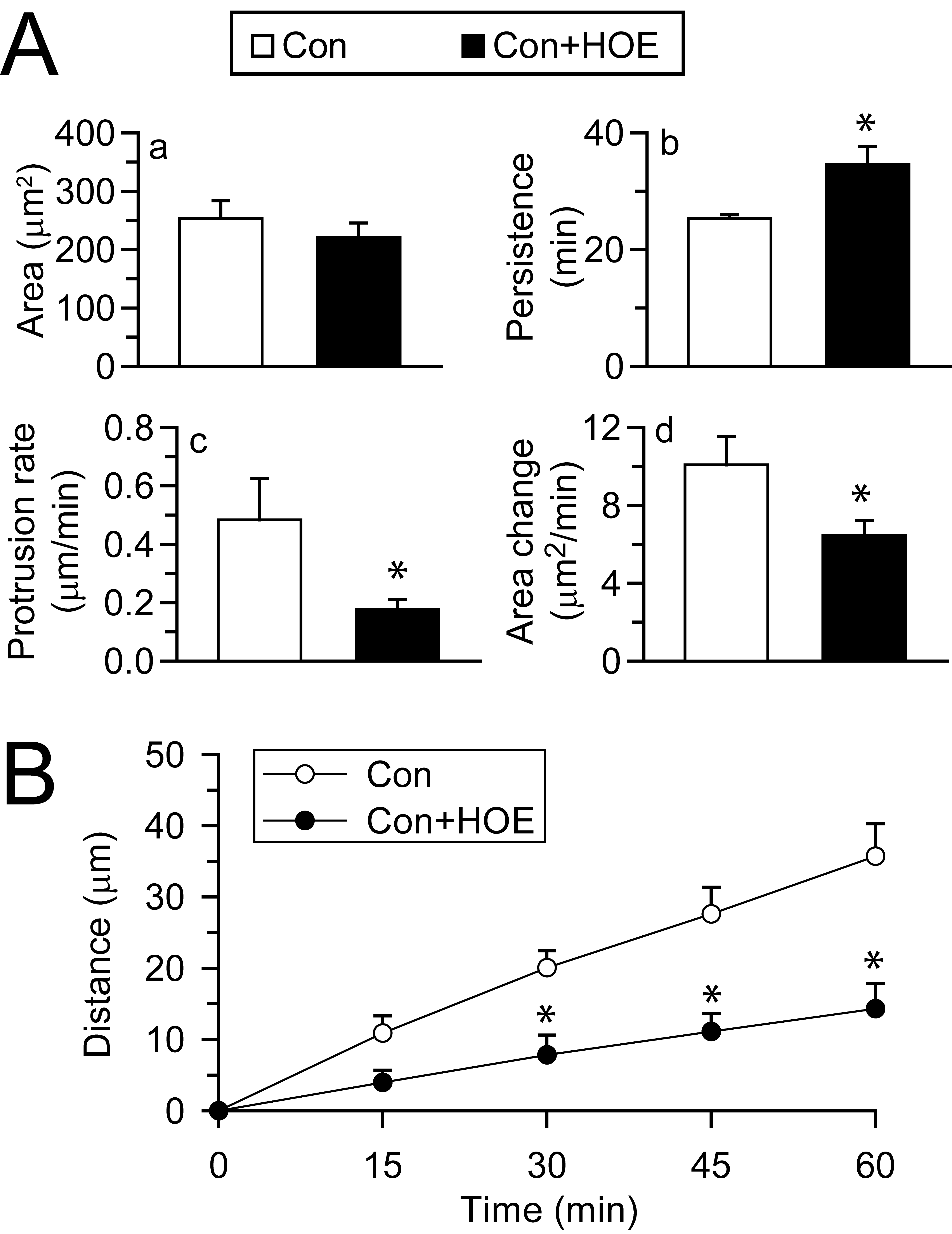

Supplement: Figure S2 — NHE-1 is involved in basal movement of microglia. BV2 microglial movement was monitored for 60 min in the presence or absence of 1 µM HOE 642. A. Summarized lamellipodial area (a), persistence (b), protrusion rate (c) and area change (d) were shown. B. Accumulated moving distance during 0-60 min was shown. Data were mean ± SEM. n =3 independent cultures. * p < 0.05 vs. Con. (TIF) [file pone.0074201.s002.tif]

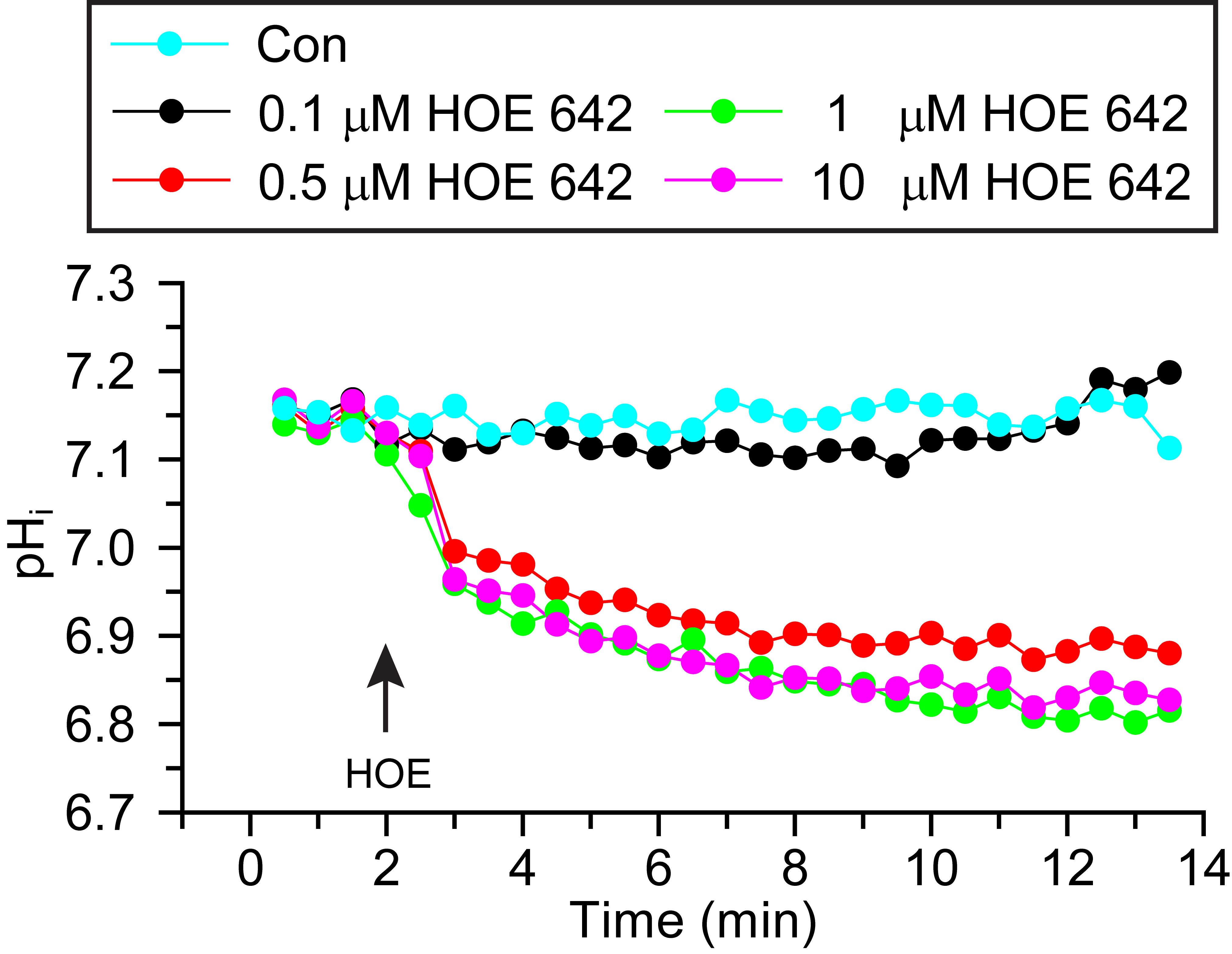

Supplement: Figure S3 — Inhibition of NHE-1 with HOE-642 abolishes pHi regulation in BV2 microglia in a dose-dependent manner. BV2 cells were loaded with 1.5 µM BCECF and monitored for 14 min. HOE 642 was added at 2 min. Traces of BV2 cell body pHi in response to 0, 0.1, 0.5, 1, or 10 µM of HOE 642 were shown. (TIF) [file pone.0074201.s003.tif]

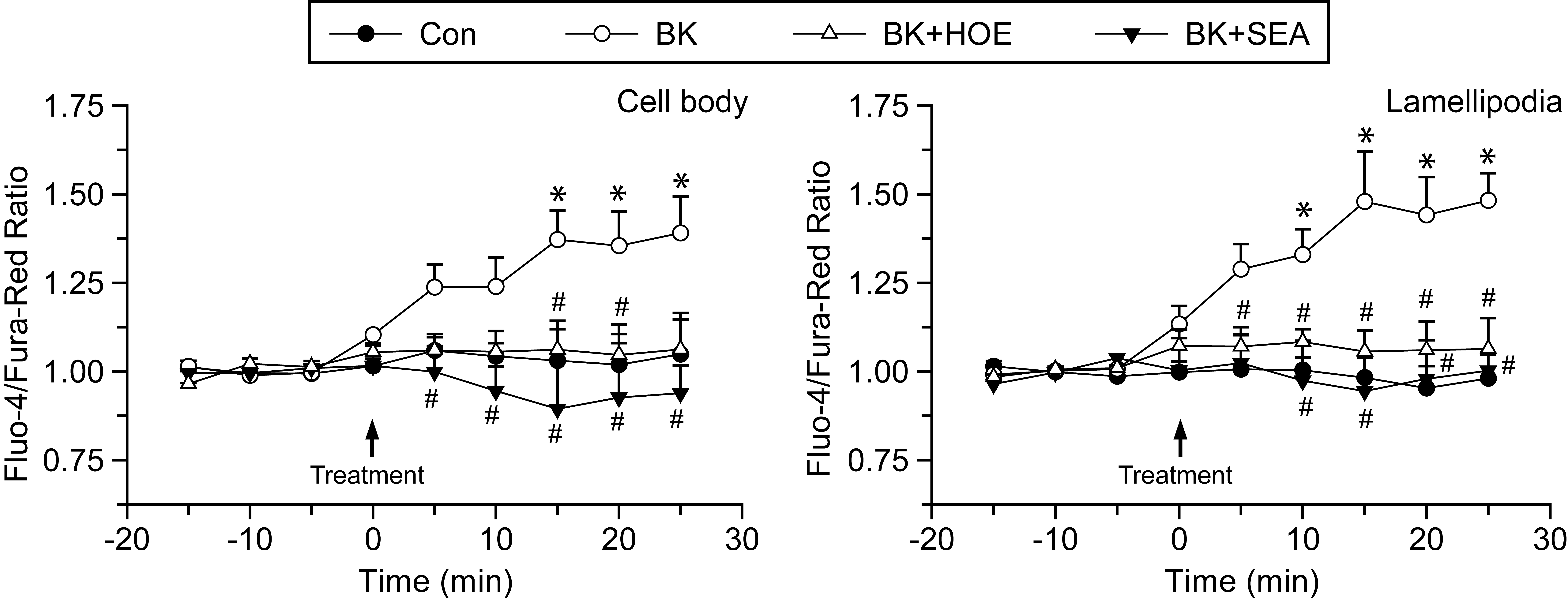

Supplement: Figure S4 — BK-mediated elevation of intracellular Ca2+ in primary microglia depends on concurrent activation of NHE-1 and NCXrev. Summarized data of BK-induced intracellular Ca2+ elevation in primary microglial cell body (left panel) and lamellipodia (right panel) were shown. 300 nM BK was used to induce intracellular Ca2+ elevation. For HOE and SEA treatment, 1 µM HOE 642 or 1 µM SEA 0400 was given together with 300 nM BK. Inhibition of NHE-1 or NCXrev activity with HOE 642 or SEA 0400 abolished Ca2+ i elevation induced by BK. Data were mean ± SEM (n = 3 independent cultures, data were calculated from 9–12 cells for each group). * p < 0.05 vs. corresponding Con; # p < 0.05 vs. corresponding BK. (TIF) [file pone.0074201.s004.tif]

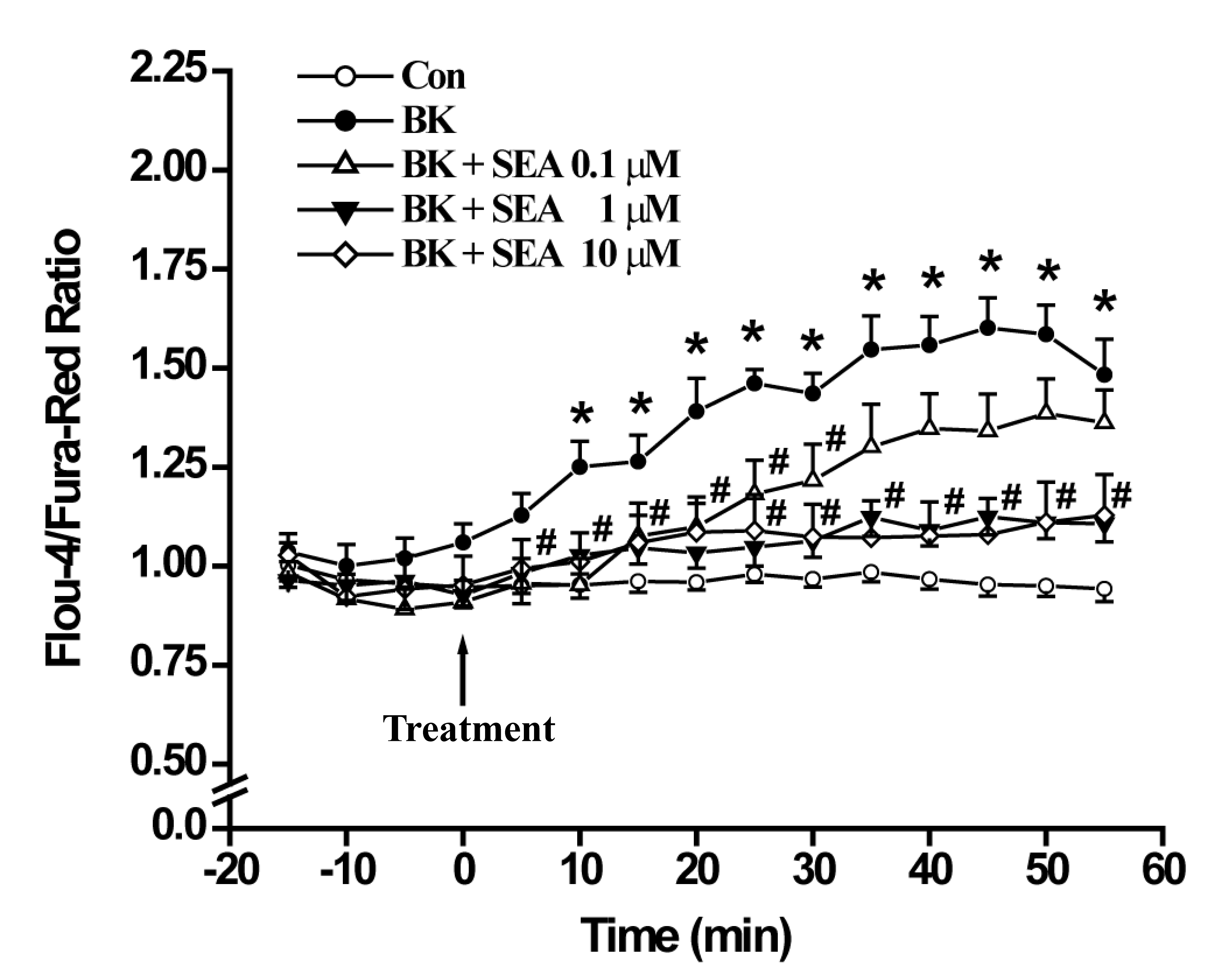

Supplement: Figure S5 — NCXrev functions in BK-mediated elevation of intracellular Ca2+ in BV2 cells. Summarized data of SEA0400 dose-dependent blockade of Ca2+ i elevation in BV2 cells were shown. For SEA treatment, 0.1, 1 or 10 µM SEA 0400 was used. Data were mean ± SEM (n = 4-6 independent cultures, data were calculated from 12-19 cells for each group). * p < 0.05 vs. corresponding Con; # p < 0.05 vs. corresponding BK. (TIF) [file pone.0074201.s005.tif]

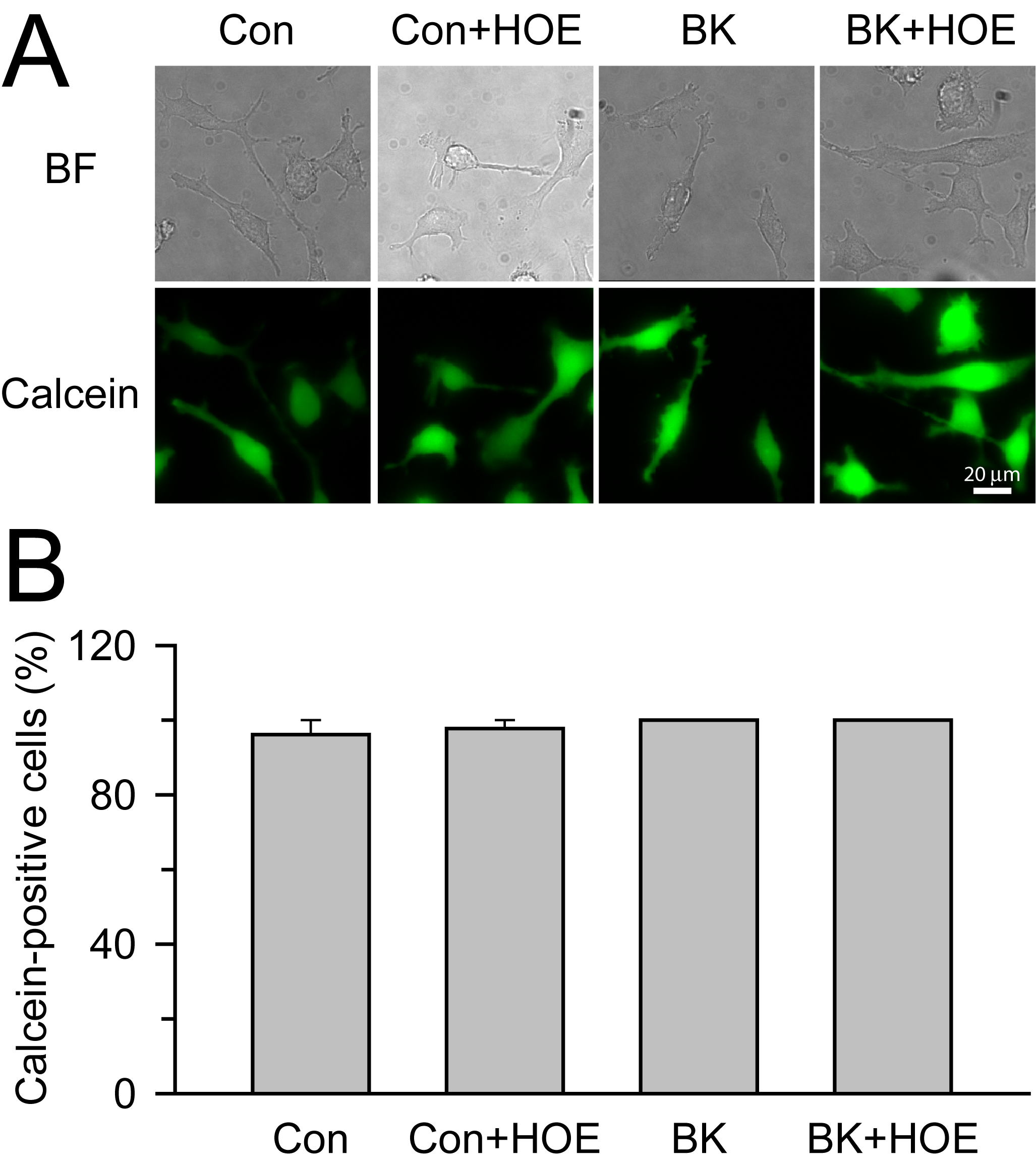

Supplement: Figure S6 — Evaluation of BV2 microglial viability during time-lapse motility monitoring. BV2 microglia were loaded with calcein AM (0.5 µM) for 30 min prior to the 60-min time-lapse imaging as described in Methods. A. Representative images of BV2 cells under brightfield (BF) or calcein AM loaded BV2 cells after 60 min of treatment were shown: control (Con), 1 µM HOE 642 (Con+HOE), 300 nM BK (BK), or 300 nM BK + 1 µM HOE 642 (BK+HOE). Scale bar: 20 µm. B. Calcein-poistive cells were counted in each group and normalized with total cells shown under brightfield. Data were mean ± SEM. n = 2 independent cultures. (TIF) [file pone.0074201.s006.tif]

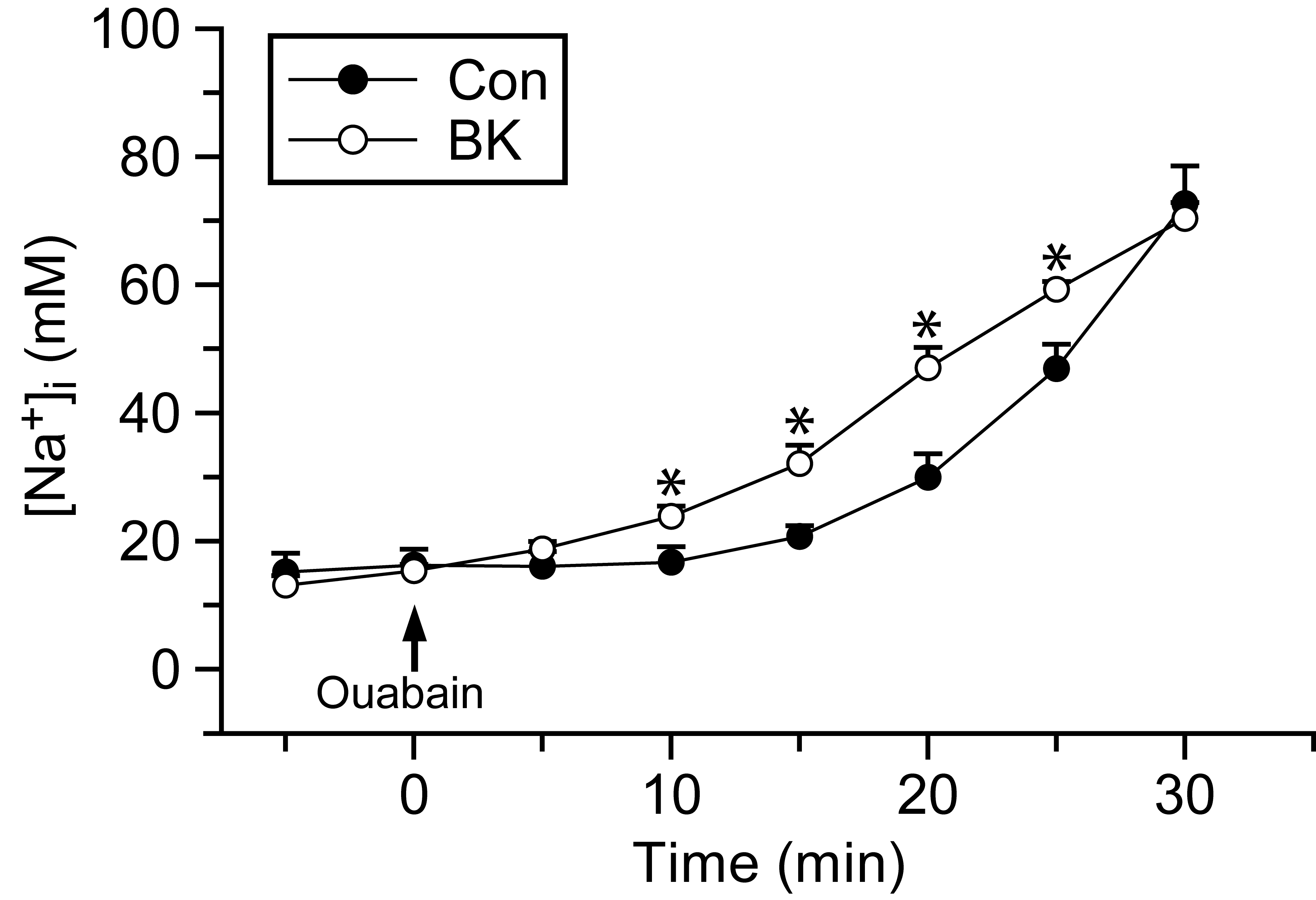

Supplement: Figure S7 — Inhibition of Na+–K+-ATPase function unmasks intracellular Na+ overload in microglia. Summarized data of [Na+]i in primary microglial cell body were shown. 0.5 mM ouabain was added to inhibit Na+-K+-ATPase activity in the absence or the presence of 300 nM BK. BK induced a faster increase in [Na+]i in the presence of ouabain. Data are mean ± SEM (n = 4 independent cultures, data were calculated from 12-16 cells for each group). * p < 0.05 vs. corresponding Con. (TIF) [file pone.0074201.s007.tif]
